# Building consensus around the assessment and interpretation of Symbiodiniaceae diversity

**DOI:** 10.7717/peerj.15023

**Published:** 2023-05-02

**Authors:** Sarah W. Davies, Matthew H. Gamache, Lauren I. Howe-Kerr, Nicola G. Kriefall, Andrew C. Baker, Anastazia T. Banaszak, Line Kolind Bay, Anthony J. Bellantuono, Debashish Bhattacharya, Cheong Xin Chan, Danielle C. Claar, Mary Alice Coffroth, Ross Cunning, Simon K. Davy, Javier del Campo, Erika M. Díaz-Almeyda, Jörg C. Frommlet, Lauren E. Fuess, Raúl A. González-Pech, Tamar L. Goulet, Kenneth D. Hoadley, Emily J. Howells, Benjamin C. C. Hume, Dustin W. Kemp, Carly D. Kenkel, Sheila A. Kitchen, Todd C. LaJeunesse, Senjie Lin, Shelby E. McIlroy, Ryan McMinds, Matthew R. Nitschke, Clinton A. Oakley, Raquel S. Peixoto, Carlos Prada, Hollie M. Putnam, Kate Quigley, Hannah G. Reich, James Davis Reimer, Mauricio Rodriguez-Lanetty, Stephanie M. Rosales, Osama S. Saad, Eugenia M. Sampayo, Scott R. Santos, Eiichi Shoguchi, Edward G. Smith, Michael Stat, Timothy G. Stephens, Marie E. Strader, David J. Suggett, Timothy D. Swain, Cawa Tran, Nikki Traylor-Knowles, Christian R. Voolstra, Mark E. Warner, Virginia M. Weis, Rachel M. Wright, Tingting Xiang, Hiroshi Yamashita, Maren Ziegler, Adrienne M. S. Correa, John Everett Parkinson

**Affiliations:** 1 Department of Biology, Boston University, Boston, MA, United States; 2 Department of Integrative Biology, University of South Florida, Tampa, FL, United States; 3 Department of BioSciences, Rice University, Houston, TX, United States; 4 Rosenstiel School of Marine, Atmospheric, and Earth Science, University of Miami, Miami, FL, United States; 5 Unidad Académica de Sistemas Arrecifales, Universidad Nacional Autónoma de México, Puerto Morelos, Mexico; 6 Australian Institute of Marine Science, Townsville, Australia; 7 Department of Biological Sciences, Florida International University, Miami, FL, United States; 8 Department of Biochemistry and Microbiology, Rutgers University, New Brunswick, NJ, United States; 9 Australian Centre for Ecogenomics, School of Chemistry and Molecular Biosciences, The University of Queensland, Brisbane, QLD, Australia; 10 Nearshore Habitat Program, Washington State Department of Natural Resources, Olympia, WA, USA; 11 Department of Geology, University at Buffalo, Buffalo, NY, United States; 12 Daniel P. Haerther Center for Conservation and Research, John G. Shedd Aquarium, Chicago, IL, United States; 13 School of Biological Sciences, Victoria University of Wellington, Wellington, New Zealand; 14 Institut de Biologia Evolutiva (CSIC - Universitat Pompeu Fabra), Barcelona, Catalonia, Spain; 15 Department of Natural Sciences, New College of Florida, Sarasota, FL, United States; 16 Centre for Environmental and Marine Studies, Department of Biology, University of Aveiro, Campus Universitário de Santiago, Aveiro, Portugal; 17 Department of Biology, Texas State University, San Marcos, TX, United States; 18 Department of Biology, Pennsylvania State University, State College, PA, United States; 19 Department of Biology, University of Mississippi, University, MS, United States; 20 Department of Biological Sciences, University of Alabama—Tuscaloosa, Tuscaloosa, AL, United States; 21 National Marine Science Centre, Faculty of Science and Engineering, Southern Cross University, Coffs Harbour, NSW, Australia; 22 Department of Biology, University of Konstanz, Konstanz, Germany; 23 Department of Biology, University of Alabama—Birmingham, Birmingham, Al, United States; 24 Department of Biological Sciences, University of Southern California, Los Angeles, CA, United States; 25 Division of Biology and Biological Engineering, California Institute of Technology, Pasadena, CA, United States; 26 Department of Biology, Pennsylvania State University, University Park, PA, United States; 27 Department of Marine Sciences, University of Connecticut, Mansfield, CT, United States; 28 Swire Institute of Marine Science, School of Biological Sciences, The University of Hong Kong, Pok Fu Lam, Hong Kong; 29 Center for Global Health and Infectious Disease Research, University of South Florida, Tampa, FL, United States; 30 Red Sea Research Center (RSRC), Division of Biological and Environmental Science and Engineering (BESE), King Abdullah University of Science and Technology, Thuwal, Saudi Arabia; 31 Department of Biological Sciences, University of Rhode Island, Kingston, RI, United States; 32 Minderoo Foundation, Perth, WA, Australia; 33 Department of Biology, Chemistry and Marine Sciences, Faculty of Science, University of the Ryukyus, Nishihara, Okinawa, Japan; 34 The Cooperative Institute For Marine and Atmospheric Studies, Miami, FL, United States; 35 Department of Biological Oceanography, Red Sea University, Port-Sudan, Sudan; 36 School of Biological Sciences, The University of Queensland, St. Lucia, QLD, Australia; 37 Department of Biological Sciences, University at Buffalo, Buffalo, NY, United States; 38 Marine Genomics Unit, Okinawa Institute of Science and Technology Graduate University, Okinawa, Japan; 39 School of Life Sciences, University of Warwick, Coventry, UK; 40 School of Environmental and Life Sciences, University of Newcastle, Callaghan, NSW, Australia; 41 Department of Biology, Texas A&M University, College Station, TX, United States; 42 Climate Change Cluster, University of Technology Sydney, Ultimo, NSW, Australia; 43 Department of Marine and Environmental Science, Nova Southeastern University, Dania Beach, FL, United States; 44 Department of Biology, University of San Diego, San Diego, CA, United States; 45 School of Marine Science and Policy, University of Delaware, Lewes, DE, United States; 46 Department of Integrative Biology, Oregon State University, Corvallis, OR, United States; 47 Department of Biological Sciences, Southern Methodist University, Dallas, TX, United States; 48 Fisheries Technology Institute, Japan Fisheries Research and Education Agency, Ishigaki, Okinawa, Japan; 49 Department of Animal Ecology & Systematics, Justus Liebig University Giessen (Germany), Giessen, Germany

**Keywords:** Symbiodiniaceae, Symbiosis, ITS2, Coral, Cnidarian, Species, Population, Community, Genetic diversity, Collaborative

## Abstract

Within microeukaryotes, genetic variation and functional variation sometimes accumulate more quickly than morphological differences. To understand the evolutionary history and ecology of such lineages, it is key to examine diversity at multiple levels of organization. In the dinoflagellate family Symbiodiniaceae, which can form endosymbioses with cnidarians (*e.g*., corals, octocorals, sea anemones, jellyfish), other marine invertebrates (*e.g.*, sponges, molluscs, flatworms), and protists (*e.g*., foraminifera), molecular data have been used extensively over the past three decades to describe phenotypes and to make evolutionary and ecological inferences. Despite advances in Symbiodiniaceae genomics, a lack of consensus among researchers with respect to interpreting genetic data has slowed progress in the field and acted as a barrier to reconciling observations. Here, we identify key challenges regarding the assessment and interpretation of Symbiodiniaceae genetic diversity across three levels: species, populations, and communities. We summarize areas of agreement and highlight techniques and approaches that are broadly accepted. In areas where debate remains, we identify unresolved issues and discuss technologies and approaches that can help to fill knowledge gaps related to genetic and phenotypic diversity. We also discuss ways to stimulate progress, in particular by fostering a more inclusive and collaborative research community. We hope that this perspective will inspire and accelerate coral reef science by serving as a resource to those designing experiments, publishing research, and applying for funding related to Symbiodiniaceae and their symbiotic partnerships.

## Introduction

Dinoflagellates in the family Symbiodiniaceae occupy multiple ecological niches on tropical, subtropical, and temperate reefs, ranging from species that are exclusively free-living to those that form symbioses with marine invertebrates (LaJeunesse et al., 2018). The biology of symbiotic Symbiodiniaceae has been a major research focus due to the integral role these mutualists play in the health of scleractinian corals and other marine invertebrates (Glynn, 1996; Hughes et al., 2017). Although many scleractinian coral species exhibit specificity for particular Symbiodiniaceae (Baker, 2003; Hume et al., 2020; Thornhill et al., 2014), some coral species and even individual coral colonies can associate with a diversity of algal symbionts (Baker & Romanski, 2007; Silverstein, Correa & Baker, 2012). Moreover, not all host-symbiont pairings are equally resistant or resilient to stress (Abrego et al., 2008; Berkelmans & Van Oppen, 2006; Hoadley et al., 2019; Howells et al., 2013a; Sampayo et al., 2008), and a change in symbiont community may enhance tolerance to future stress. Thus, efforts to characterize the genetic and functional diversity within Symbiodiniaceae not only advances our fundamental knowledge of the evolution and ecology of microeukaryotes, but also provides insights into the potential for cnidarian-Symbiodiniaceae partnerships, and ultimately for coral reefs, to respond to rapidly changing environments.

**Figure 1:**
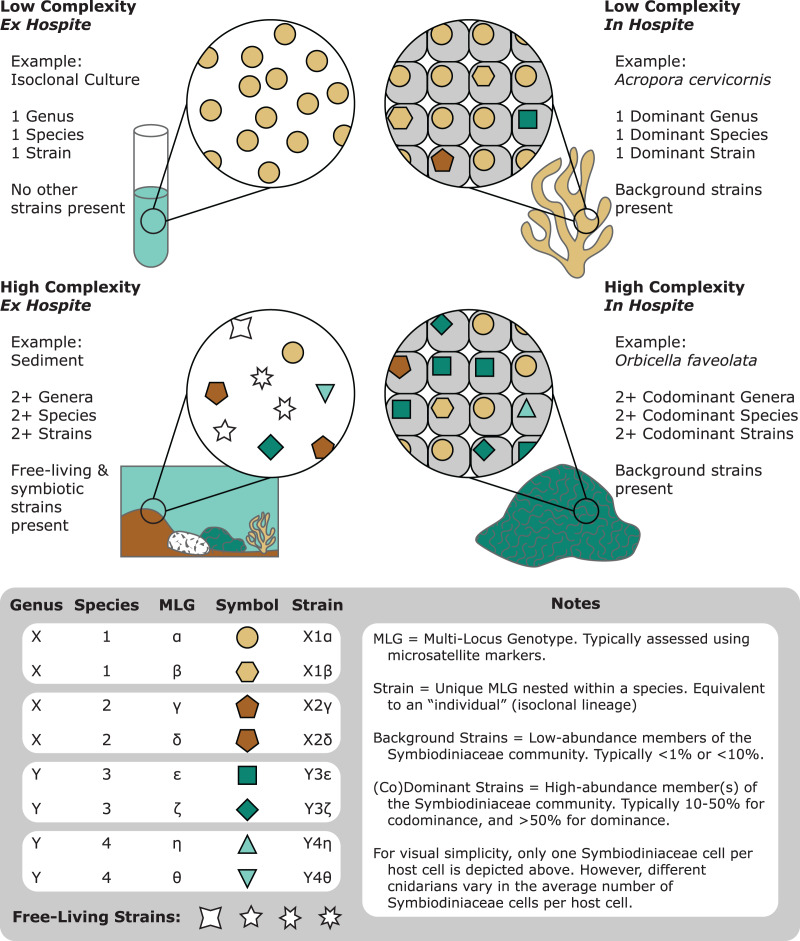
A representation of the various degrees of complexity in Symbiodiniaceae genetic diversity among different habitats (*e.g.*, cultures, corals, and sediments). Communities of Symbiodiniaceae within a given sample can encompass multiple strains, populations, species, and genera.

The first “*Symbiodinium*” species was formally described by Freudenthal (1962). As more associations with these endosymbiotic dinoflagellates were cataloged, the utility of allozymes (Schoenberg & Trench, 1980) and later ribosomal markers (LaJeunesse, 2001; Rowan & Powers, 1991) to distinguish different lineages became apparent. Continued exploration of Symbiodiniaceae diversity through molecular genetics ultimately resulted in a recent systematic revision, delineating at least eleven genera and many species (LaJeunesse et al., 2021, 2018; Nitschke et al., 2020; Pochon & LaJeunesse, 2021). However, despite numerous advances in our ability to resolve Symbiodiniaceae populations, often allowing for genus, species, or even strain level identification (Thornhill et al., 2017), diversity assessments pose substantial challenges (Fig. 1). For example, Symbiodiniaceae density often exceeds 1–2 million cells *per* square centimeter of host tissue (Fitt et al., 2000). Further, hosts may associate with a single species or a mixture of multiple species and/or genera (Baker & Romanski, 2007; Coffroth et al., 2010; Kemp, Fitt & Schmidt, 2008; Rowan & Knowlton, 1995; Thornhill et al., 2017, 2006; van Oppen et al., 2005). In addition, Symbiodiniaceae have expansive genomes (~1–5 Gbp; Saad et al., 2020), often including multi-copy genes and extensive gene duplication (Lin, 2011; González-Pech et al., 2021). Therefore, many approaches to resolve Symbiodiniaceae taxonomy rely on multi-copy gene markers. For example, the multi-copy internal transcribed spacer 2 (ITS2) rDNA region is most frequently used to resolve Symbiodiniaceae lineages, yet data generated by this marker straddle intergenomic and intragenomic variation (the latter of which is abbreviated as IGV), limiting its utility for some applications (Smith, Ketchum & Burt, 2017). This issue has fueled an active debate within the research community regarding the interpretation of ITS2 molecular data and likely contributed to underuse of other molecular markers, even though they may be more appropriate in some contexts (LaJeunesse & Thornhill, 2011; Takishita et al., 2003).

Indeed, the increasing popularity of amplicon-sequencing methods (Arif et al., 2014; Green et al., 2014; Howe-Kerr et al., 2020; Hume et al., 2019; Quigley et al., 2014), exploration of additional molecular markers (Pochon et al., 2019, 2012; Smith et al., 2020; Takabayashi, Santos & Cook, 2004), and incorporation of whole-genome datasets (Dougan et al., 2022; González-Pech et al., 2021; Liu et al., 2018) have led to novel insights into Symbiodiniaceae diversity. However, such advances have led to additional challenges. For example, most genetic loci exhibit differential utility across Symbiodiniaceae genera (Pochon, Putnam & Gates, 2014). Furthermore, different analytical pipelines and thresholds applied to the same marker(s) among studies have led to different estimates of genetic variation and interpretation of their functional importance (Cunning, Gates & Edmunds, 2017; Howells et al., 2016; Wham & LaJeunesse, 2016; Wirshing & Baker, 2016). These issues have further fueled the debate around which markers to use and how to interpret the resulting data.

Recognizing that continued debate may complicate the process of scientific inquiry, we sought to identify areas of consensus regarding the assessment and interpretation of Symbiodiniaceae genetic diversity. Sixty-one scientists from 12 countries, spanning expertise in the taxonomy, physiology, genomics, and ecology of Symbiodiniaceae and other marine microbes, participated in a workshop funded by the National Science Foundation titled “Building consensus around the quantification and interpretation of Symbiodiniaceae diversity,” held virtually in July 2021. The overall aim was to reduce barriers to those designing experiments, publishing research, and applying for funding related to Symbiodiniaceae and their partnerships. The major workshop outcomes are summarized herein, though not exhaustively. We highlight techniques that are broadly accepted by many experts in the field and point out caveats and considerations for these approaches (Box 1). Where agreement was not reached, we identify the key issues that remain unresolved and point to technologies that might help fill knowledge gaps so that consensus can be achieved in the future. We conclude with suggestions for how to make the Symbiodiniaceae research community a more inclusive and welcoming space that promotes innovation as we navigate the coral reef crisis. Above all, we wish to stress that the choice of genetic marker(s) and analytical framework(s) for interpreting Symbiodiniaceae diversity will always depend on the research question at hand, along with the availability of resources (*e.g.*, for sample preservation, processing, and computation), and that these options will inevitably evolve as our understanding of the system continues to develop.

**Box 1:** Major workshop outcomes and consensus highlights.

**General**
Different research questions require different levels of resolution of Symbiodiniaceae diversity (*e.g*. species, populations, communities). Molecular markers evolve at different rates and vary in their ability to resolve different Symbiodiniaceae taxonomic levels and lineages, requiring careful selection of the appropriate marker(s) for a given question. Many markers and analytical approaches are available, each with their own strengths and weaknesses. As genomic resources for Symbiodiniaceae continue to be developed and technologies advance, so will options for analyzing and interpreting diversity. Collaborations among research groups can ameliorate methodological and analytical disconnect within the Symbiodiniaceae community, while also reducing costs associated with answering complex and integrative research questions.
**1. Species-Level Assessment of Symbiodiniaceae Diversity**
Resolving Symbiodiniaceae to the species level is important. Species identification forms the basis of comparative physiological, ecological, and evolutionary investigations within Symbiodiniaceae. A robust Symbiodiniaceae taxonomy is required to facilitate scientific communication, link past and future research, and establish legal frameworks for conservation. Funding to develop and maintain up-to-date public taxonomic tools and databases should be increased. There are distinctions between *describing* a new species, *recognizing* a new species, and *identifying* a known species. Reef researchers benefit from incorporation and consideration of the current taxonomy whenever possible. Expanding publicly accessible Symbiodiniaceae culture collections and their formal genetic, morphological, and physiological description will drive taxonomic, ecological, physiological, and genomic research. Supporting these resources for use by the scientific community should be a priority for long-term funding.
**2. Population-Level Assessment of Symbiodiniaceae**
Population-level studies evaluate the distribution of genetic variation within Symbiodiniaceae species, often across spatiotemporal gradients or among host taxa, to understand the influence of evolutionary processes such as gene flow, genetic drift, and natural selection. When multiple Symbiodiniaceae lineages are present within host colonies, population-level questions are more challenging to address. Pre-screening to determine which lineages are present within samples is necessary to determine the marker(s) needed to address population-level questions in Symbiodiniaceae. Microsatellite loci can be effective at addressing population-level questions in Symbiodiniaceae if used appropriately. The ITS2 region of Symbiodiniaceae rDNA may be an effective marker for distinguishing between different populations, but requires thorough validation to distinguish intra- and inter-genomic variation.
**3. Community-Level Assessment of Symbiodiniaceae**
Symbiodiniaceae communities can be conceptualized at different scales. The presence of two or more Symbiodiniaceae species within a host individual constitutes a “local community.” Symbiodiniaceae diversity at larger scales (*e.g.*, among conspecific host colonies, multiple host species, or across environmental pools such as sediments and the water column including free-living Symbiodiniaceae) constitutes a “macroscale community.” The total diversity of both local and macroscale communities is likely underestimated. Local Symbiodiniaceae communities are often composed of representatives of different genera, rather than multiple species or lineages within the same genus. Marker genes that exhibit inter- and intra-genomic variation (as well as variation in copy number across lineages) make it challenging to characterize Symbiodiniaceae community composition. Quantifying this molecular variation for Symbiodiniaceae genera and species is a priority. The Symbiodiniaceae ITS2 marker can be useful for describing Symbiodiniaceae communities but there are circumstances where multiple markers or other approaches may be more appropriate. The majority of researchers at the workshop reported greatest familiarity and comfort with the ITS2 marker, which may have contributed to its popularity in characterizing Symbiodiniaceae communities. There is a lack of consensus regarding best practices for interpreting Symbiodiniaceae gene amplicon data to identify species, and for applying and interpreting community diversity metrics. Authors are encouraged to clearly highlight assumptions associated with their data interpretation, acknowledge that other interpretations exist, and discuss whether or not alternative interpretations change the biological or ecological findings of their study.
**4. Beyond Genotype: Phenotyping Symbiodiniaceae**
Phenotypic diversity varies greatly within and between Symbiodiniaceae species, thus it is critical to avoid overestimating the functional significance of a given symbiont based on taxonomic assignment alone (*e.g.*, assuming that all *Durusdinium* spp. are heat-tolerant). There is a need to develop technologies to functionally assess Symbiodiniaceae in culture, *in hospite*, and in the environment–and to better contextualize the resulting phenotypes–with the understanding that functional diversity will vary depending on the metrics used. When attempting to understand phenotypic variability among strains and species, using cultures of Symbiodiniaceae can help control confounding variables. However, because cultures are, by nature, artificial environments, performance *in vitro* may differ from performance *in hospite*, and many species are difficult to culture.
**5. Integrating Multiomic Technologies to Study Symbiodiniaceae**
Various ’omics techniques have been used to address Symbiodiniaceae taxonomic, functional, and physiological research questions. Because each technique has unique considerations, leveraging these novel tools requires stringent ground-truthing and the development of quality standards. Genome projects have improved tremendously over the past decade, but there are unique biological obstacles that have restricted Symbiodiniaceae genome assembly quality. Examples include large genome sizes, high repeat content, and difficulty annotating gene functions. Integrating multiple techniques, such as transcriptomics and proteomics, and coupling these with phenotyping methods, can help answer outstanding questions regarding Symbiodiniaceae-host interactions. Efficient experimentation will require combining expertise across laboratories.
**6. Ensuring an Inclusive Symbiodiniaceae Research Community**
Critical examination is at the heart of scientific inquiry. A diversity of perspectives has always been and will continue to be needed to move the Symbiodiniaceae field forward. The publication process should be equitable. Recommendations to journals and scientific societies include increasing diversity on relevant editorial boards, scaling publication costs for researchers employed in countries with lower income economies, and implementing double-blind review. Researchers should actively cite articles led by diverse colleagues. Parachute science should be avoided. Recommendations include fostering long-term international collaborations and exchange programs to involve local scientists in Symbiodiniaceae research, improving sensitivity to the challenges facing colleagues in funding-limited partner institutions, and extending full collaborative benefits including authorship and grant writing opportunities to these colleagues. Accessibility and collaboration should be fostered. Recommendations include expanding a recently established database of Symbiodiniaceae researchers and their research products, maintaining hybrid format options for conferences, and supporting long-term funding for international collaborations. It is critical to improve recruitment, retention, and promotion of scholars of diverse backgrounds. Recommendations include working actively to increase diversity at all levels of academia and science, promoting the work of minority scientists, and providing strong multidimensional mentorship to support and retain these scientists throughout each career stage.

## Guidance for species-level assessment of symbiodiniaceae diversity

### Why is species-level resolution important for Symbiodiniaceae?

Species are evolutionarily independent lineages and therefore represent a fundamental level of biological organization. Species-level resolution provides insight into the ecological and evolutionary mechanisms that create diversity, and forms the basis of comparative physiological investigations (Kareiva & Levin, 2015). The delineation of species can affect nearly all scales of inquiry, from biochemical pathways to ecosystem processes. Species-level diversity in Symbiodiniaceae has been discussed in the literature since the description of *Symbiodinium microadriaticum* in 1962 by Freudenthal (1962). As more diversity was uncovered and more species were recognized (LaJeunesse, 2001; LaJeunesse & Trench, 2000; Rowan & Powers, 1992, 1991; Schoenberg & Trench, 1976; Trench & Blank, 1987), controversy arose as to where to draw species boundaries (Apprill & Gates, 2007; Correa & Baker, 2009; Cunning, Glynn & Baker, 2013; LaJeunesse et al., 2014; LaJeunesse, Parkinson & Reimer, 2012; Stat et al., 2012; Thornhill, LaJeunesse & Santos, 2007; Wham & LaJeunesse, 2016). At present, there is general consensus among Symbiodiniaceae specialists about the need for species-level resolution, as well as support for current taxonomic methodologies that are underpinned by genetic, ecological, and morphological data (LaJeunesse et al., 2018; Voolstra et al., 2021b). Such taxonomic descriptions facilitate scientific communication and are necessary for establishing legal frameworks for conservation (IUCN, 2021). The recent elevation of most Symbiodiniaceae “Clades” to genera has provided some clarity (LaJeunesse et al., 2021, 2018; Nitschke et al., 2020; Pochon & LaJeunesse, 2021), but the small number of formal species-level descriptions for the large genetic diversity found within most Symbiodiniaceae genera constitutes a formidable barrier to progress.

Without robust species delineation, functional differences can inadvertently be ascribed to incorrect taxonomic levels or non-existent biological entities. For example, the genus level may be too coarse and lead to over-generalizations regarding the physiology or function of Symbiodiniaceae variants (see “Beyond Genotype: Phenotyping Symbiodiniaceae”). A statement such as “*the genus* Cladocopium *consists of heat-sensitive species*” overlooks the superior stress tolerance of some *Cladocopium* species, including the dominance of *Cladocopium thermophilum* in corals on some of the world’s hottest reefs in the Persian/Arabian Gulf (Abrego et al., 2008; Hume et al., 2015; Varasteh et al., 2018). However, diversity assessments based on gene sequence variants may recover both interspecific variation (resolving distinct species) and intraspecific variation (sequence diversity within a single genome). This is a major issue for the commonly used multi-copy ITS2 gene. Consequently, a statement such as “*Symbiodiniaceae harboring the ITS2 D13 sequence variant are adapted to temperate environments*” overlooks the fact that ITS2 sequence variants D8, D8–12, D12–13, and D13 are all characteristic of the same species, *Durusdinium eurythalpos* (LaJeunesse et al., 2014). The statement could give a false impression that entities harboring the D8 variant are phylogenetically and ecologically distinct from those harboring D13. In this scenario, because we know the ITS2 profile of *D. eurythalpos*, we can clarify that the four sequence variants belong to the same species. However, for many undescribed species, the profiles are not yet resolved. Such issues are problematic because they may confuse ecological interpretations of sequence data, particularly in datasets composed of communities of different symbiont species where some consist of overlapping ITS2 intragenomic variants.

### What types of data can identify Symbiodiniaceae species?

Although taxonomic descriptions are fundamental, *describing* a new species is not the same as *recognizing* a new species or *identifying* a known species. *Describing* should be based on multiple lines of evidence, whereas *recognizing* or *identifying* may require generating and interpreting data from only one or two diagnostic methods. At minimum, there are six major components of a valid Symbiodiniaceae species description: (1) information on at least two congruent genes (see our recommendations below in “How can we Resolve Symbiodiniaceae Species with Genetic Markers?”), (2) comparison of genetic data against that from other Symbiodiniaceae, (3) morphological description (*e.g.*, comparison of cell size measurements against that from other Symbiodiniaceae), (4) a holotype or name-bearing type specimen (at minimum, an image of cells under light microscopy, but preserved cells are preferable), (5) deposition of the type specimen in a permanent archive (*e.g.*, a museum or herbarium for preserved cells, but if only images are available, their publication in a peer-reviewed journal is sufficient), and (6) proposition of a valid name (according to the International Code of Nomenclature for Algae, Fungi, and Plants; Turland et al., 2018). Where possible, ecological descriptions such as host associations and biogeographic ranges are also encouraged, although sometimes such information is not available.

**Figure 2:**
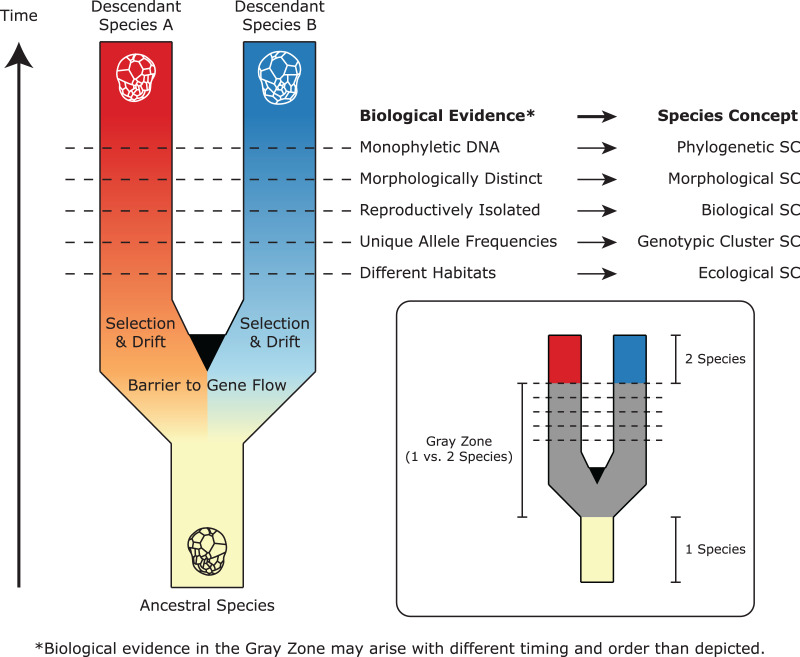
A simplified representation of Symbiodiniaceae speciation, species concepts (SC), and associated biological evidence. In this example, one ancestral species splits and diverges to become two descendant lineages after barriers to gene flow are established. Through selection and drift, these lineages evolve different properties, which satisfy the criteria of different species concepts (represented by horizontal lines). Because these properties may arise at different times and in different orders, there is a “gray zone” where conflict among species concepts may result in controversy about whether one or two species exist. Modified from De Queiroz (2007) and Leliaert et al. (2014).

The Biological Species Concept dictates that if two organisms cannot reproduce and create viable offspring, they should be considered different species (Mayr, 1942). Unfortunately, it has been impossible to apply this criterion to Symbiodiniaceae, as no direct observation of sexual reproduction has been made to date (but see Figueroa, Howe-Kerr & Correa, 2021; Shah et al., 2020). Fortunately, many other species concepts exist, each placing emphasis on different criteria (De Queiroz, 2007; Leliaert et al., 2014). Robust species descriptions satisfy multiple species concepts using independent lines of evidence. For Symbiodiniaceae, the field has largely applied three key types of data: morphological (cell size and cell wall features), ecological (host specificity and biogeographic distribution), and phylogenetic (divergence across multiple DNA markers), along with the assignment of type material (Fig. 2). The taxonomic framework for describing species has matured since the earliest effort by Freudenthal (1962). For example, in line with the Morphological Species Concept, Trench & Blank (1987) proposed three new species based on Symbiodiniaceae cell ultrastructure. They used transmission electron microscopy (TEM) to reveal features such as the nucleus, chromosomes, pyrenoid, chloroplast thylakoid membranes, and cell size; additionally, they used scanning electron microscopy (SEM) to observe thecal plates and the arrangement of the two flagella. Technological advancements in SEM resolution now enable complete morphological characterization of amphiesmal vesicles in the cell wall (Jeong et al., 2014; LaJeunesse, Lee & Gil-Agudelo, 2015; Lee, Jeong & LaJeunesse, 2020; Lee et al., 2015; Nitschke et al., 2020), though such plate tabulations tend to be variable within species (LaJeunesse et al., 2018).

As an increasing number of host species are sampled, it has become clear that the Ecological Species Concept can also be used to support Symbiodiniaceae species descriptions. Although not diagnostic in all cases (Cunning, Glynn & Baker, 2013), symbiosis ecology can be particularly useful for Symbiodiniaceae species that exhibit host-specificity or coadaptation with their hosts (Davies et al., 2020; Finney et al., 2010; Howells et al., 2020; Santos et al., 2004; Smith, Ketchum & Burt, 2017; Thornhill et al., 2014). For example, *Cladocopium pacificum* and *Cladocopium latusorum* are found exclusively within corals of the genus *Pocillopora* (Turnham et al., 2021). Ultimately, because Symbiodiniaceae do not always have distinct morphological characteristics, nor do they always exhibit host specificity, the collection of genetic data to satisfy the Phylogenetic Species Concept has also become a necessity in the description of species (see “How can we Resolve Symbiodiniaceae Species with Genetic Markers?”).

Researchers who do not endeavor to describe Symbiodiniaceae species can encourage and incentivize those who do by accurately treating taxa names as hypotheses and citing the work of taxonomists at the first mention of previously described taxa within manuscripts. We encourage incorporating existing taxonomy (*i.e.*, species names) into current research whenever possible. Due to a general lack of funding for taxonomic descriptions, formal species names are not always available for a given entity, and therefore accommodating sequence variant terminology in the literature will continue to be important. Providing synonyms (*e.g.*, the ITS2 sequence variant *and* its species name) when a species is first mentioned will improve clarity. Ensuring that community resources consolidate current and past taxonomic assignments will be challenging, but it is critical for connecting historical and future research. Guidance for vouchering Symbiodiniaceae genomic data sets from cultures or holobiont tissues has recently been put forth (Voolstra et al., 2021b). Minimum recommendations include (but are not limited to) high quality DNA voucher material, comprehensive metadata, and common phylogenetic marker sequences. Work is underway to develop a robust ‘Rosetta Stone’ that can translate between different marker designations and species names. Such efforts should be expanded in the future (*e.g.*, through incorporation into analysis pipelines for molecular data) to better facilitate efficient Symbiodiniaceae identification in complex samples.

### How can we resolve Symbiodiniaceae species with genetic markers?

No single marker is likely able to distinguish species across all Symbiodiniaceae genera reliably (Table 1). Instead, ecological and physiological studies will benefit from adopting a multi-gene approach where possible, given funding and resource limitations (see “Guidance for Community-Level Assessment of Symbiodiniaceae”). Congruence among sequence data from different cellular compartments (nuclear, chloroplast, and mitochondrial; Table 1) indicates that classifying Symbiodiniaceae using a lineage-based species concept is achievable (De Queiroz, 2007; LaJeunesse & Thornhill, 2011; Sampayo, Dove & LaJeunesse, 2009). This multi-gene approach, supported with ecological, morphological, and sometimes physiological data, has led to the formal description (or re-validation) of 39 Symbiodiniaceae species in 11 genera thus far (Table 1; Hume et al., 2015; Jeong et al., 2014; LaJeunesse, 2017; LaJeunesse et al., 2021, 2018; LaJeunesse, Lee & Gil-Agudelo, 2015; LaJeunesse et al., 2014; LaJeunesse, Parkinson & Reimer, 2012; Lee, Jeong & LaJeunesse, 2020; Lewis, Chan & LaJeunesse, 2019; Nitschke et al., 2020; Parkinson, Coffroth & LaJeunesse, 2015; Pochon & LaJeunesse, 2021; Ramsby et al., 2017; Turnham et al., 2021; Wham, Ning & LaJeunesse, 2017; Xiang et al., 2013). This taxonomic list will continue to grow, and recent whole-genome data already point toward the potential need for further revision of some genera and species (Dougan et al., 2022; González-Pech et al., 2021).

**Table 1:** Current list of formally described Symbiodiniaceae species and associated diagnostic information.

								Nuclear			Mito		Chloro	
**Genus** **(11 total)**	**Species** **(39 total)**	**ITS2 Type** **(majority sequence)**	**Symbiotic?**	**Cultured?**	**Host-specificity**	**Cell Size**	**Plate tabulation**	**ITS2**	**LSU**	**msat flankers**	**mtCOB**	**mtCOX1**	**cp23S**	**psbA** ^**ncr**^
*Breviolum*	*B. aenigmaticum*	–	U	Y	U	ND	X	D	D	D	ND	X	D	X
*Breviolum*	*B. antillogorgium*	B1	Y	Y	D	ND	X	ND	ND	D	ND	X	D	X
*Breviolum*	*B. dendrogyrum*	B1k, B1	Y	N	D	ND	X	D**	ND	D	ND	X	ND	D
*Breviolum*	*B. endomadracis*	B7	Y	N	D	ND	X	D	ND	D	D	X	ND	D
*Breviolum*	*B. faviinorum*	B14, B14a, B1	Y	N	ND	ND	X	D**	ND	D	ND	X	ND	D
*Breviolum*	*B. meandrinium*	B20, B1	Y	N	ND	ND	X	D**	ND	D	ND	X	ND	D
*Breviolum*	*B. minutum*	B1	Y	Y	ND	ND	X	ND	ND	D	ND	X	ND	D
*Breviolum*	*B. pseudominutum*	B1	U	Y	U	ND	X	ND	ND	D	D	X	ND	X
*Breviolum*	*B. psygmophilum*	B2	Y	Y	ND	ND	X	D	D	D	ND	X	D	X
*Cladocopium*	*C. goreaui*	C1	Y	Y	ND	Y	ND*	ND	ND	X	D	D	ND	D
*Cladocopium*	*C. infistulum*	C2	Y	Y	D	Y	ND*	D	D	X	D	D	D	X
*Cladocopium*	*C. latusorum*	C1b-c, C42a, C42a-b, C1c-ff	Y	N	D	Y	X	D	D	D	ND	X	X	D
*Cladocopium*	*C. pacificum*	C1d, C1d-t	Y	N	D	Y	X	D	D	D	ND	X	X	D
*Cladocopium*	*C. thermophilum*	C3	Y	N	ND	U	X	D	ND	X	D	D	ND	D
*Durusdinium*	*D. glynnii*	D1, D1-4-6	Y	N	ND	ND	X	D**	ND	D	ND	X	ND	D
*Durusdinium*	*D. boreum*	D15	Y	N	ND	ND	X	D	D	D	D	X	ND	X
*Durusdinium*	*D. eurythalpos*	D8, D12-13, D13	Y	N	ND	ND	X	D	D	D	ND	X	ND	X
*Durusdinium*	*D. trenchii*	D1a, D1-4, D1-4-6	Y	Y	ND	ND	X	D**	ND	D	ND	X	D	D
*Effrenium*	*E. voratum*	E1	N	Y	NA	M	M	M	M	X	M	X	M	M
*Freudenthalidium*	*Fr. endolithicum*	F3.8	U	Y	X	D	ND	R	R	X	R	X	R	X
*Freudenthalidium*	*Fr. heronense*	F3.7	U	Y	X	D	ND	R	R	X	R	X	R	X
*Fugacium*	*Fu. kawagutii*	F1	U	Y	U	M	X	M	M	X	X	X	X	X
*Gerakladium*	*G. endoclionum*	–	Y	N	D	ND	X	X	D	X	ND	X	R	R
*Gerakladium*	*G. spongiolum*	–	Y	N	D	ND	X	X	ND	X	ND	X	R	R
*Halluxium*	*H. pauxillum*	H7	U	Y	X	M	M	R	R	X	ND	X	R	X
*Miliolidium*	*M. leei*	D1.1	U	Y	U	M	X	R	R	X	R	R	X	X
*Philozoon*	*P. actiniarum*	A19	Y	N	D	D	X	D	ND	X	D	ND	ND	R
*Philozoon*	*P. adriaticum*	–	Y	N	D	D	X	ND	D	X	ND	ND	ND	R
*Philozoon*	*P. anthopleurum*	–	Y	N	D	D	X	ND	ND	X	ND	ND	ND	R
*Philozoon*	*P. balanophyllum*	–	Y	N	D	D	X	ND	ND	X	ND	D	ND	R
*Philozoon*	*P. colossum*	–	Y	N	D	D	X	ND	D	X	ND	D	D	R
*Philozoon*	*P. geddesianum*	–	Y	N	D	D	X	ND	ND	X	ND	D	D	R
*Philozoon*	*P. medusarum*	–	Y	N	D	D	X	ND	ND	X	ND	ND	ND	R
*Philozoon*	*P. paranemonium*	–	Y	N	D	D	X	ND	ND	X	ND	D	ND	R
*Symbiodinium*	*S. microadriaticum*	A1	Y	Y	ND	ND	ND	D	D	X	D	X	ND	D
*Symbiodinium*	*S. natans*	–	N	Y	U	ND	ND	D	D	X	D	X	D	X
*Symbiodinium*	*S. necroappetens*	A13	Y***	N	ND	ND	ND	D	D	X	D	X	ND	D
*Symbiodinium*	*S. pilosum*	A2	N	Y	U	ND	X	D	D	X	D	X	D	X
*Symbiodinium*	*S. tridacnidorum*	A6, A3a, A3*	Y	Y	ND	ND	ND	D**	D	X	D	X	D	X

**Notes:**

Mito, Mitochondrial; Chloro, Chloroplast; R,Resolves all species within the genus; D, Diagnostic (uniquely differentiates a particular species of the genus); ND, Not diagnostic (sequence/trait identical in two or more species); M, Measured but lacking congenerics or reference material for comparison; X, Not used in species description; U, Unknown (*e.g*., sampled from a symbiotic habitat but not necessarily likely to be the numerically dominant symbiont); Y, Yes; N, No; NA, Not Applicable; ND*, Not diagnostic of species, but lack of elongated amphiesmal vesicles is diagnostic of Cladocopium; D**, Some ITS2 sequences may be diagnostic, but others in the in the same genome may not be; Y***, Opportunistic and occurring at background levels unless host health is compromised.

For an extended version of the table that includes authentic cultured strains, synonyms, and key references for each species, see Table S1.

The rate of evolution of gene markers dictates their respective power to resolve distinct genetic entities and whether these entities are likely to represent distinct species (Table 1). In addition, genetic differentiation may vary among genera for the same marker region (Pochon, Putnam & Gates, 2014). Efforts are underway to develop a taxonomic key for Symbiodiniaceae species based on genetic and ecological data. We envision a dynamic dichotomous key that would guide users to the appropriate markers and characteristics for a particular host organism of interest, or alternatively, suggest combinations of markers and characteristics most likely to provide species-level resolution within specific sets of closely related Symbiodiniaceae. Such a key would also reduce project costs by identifying the most informative minimal set of markers.

### How many Symbiodiniaceae species exist?

The current best estimate for the total number of symbiotic Symbiodiniaceae species is in the range of hundreds based on phylogenetic (*e.g.*, ITS2) sequence variants (Thornhill et al., 2014). However, these species numbers are likely a significant underestimate because sampling efforts have mainly focused on scleractinian coral hosts living at shallow depths in tropical and subtropical waters. It will be important to continue describing Symbiodiniaceae species in non-scleractinian hosts, including other cnidarians; *e.g.*, octocorals (Goulet et al., 2017; Ramsby et al., 2014), zoantharians (Fujiwara et al., 2021; Mizuyama et al., 2020), actiniarians (Grajales, Rodríguez & Thornhill, 2016), corallimorpharians (Kuguru et al., 2008; Jacobs et al., 2022), hydrocorals (Rodríguez et al., 2019), jellyfish (Vega de Luna et al., 2019); as well as sponges (Hill et al., 2011; Ramsby et al., 2017), acoelomorph flatworms (Kunihiro & Reimer, 2018), molluscs (Baillie, Belda-Baillie & Maruyama, 2000; Banaszak, García Ramos & Goulet, 2013; Lim et al., 2019), ciliates (Mordret et al., 2016), and foraminifera (Pochon et al., 2007). Further collections from undersampled habitats and sources such as benthic sediment and rubble (Fujise et al., 2021; Nitschke et al., 2020; Sweet, 2014; Takabayashi et al., 2012), seagrasses and macroalgae (Porto et al., 2008; Yamashita & Koike, 2013), mesophotic depths (Frade et al., 2008; Goulet, Lucas & Schizas, 2019), the water column (Manning & Gates, 2008; Pochon et al., 2010; Sweet, 2014), and predator feces (Castro-Sanguino & Sánchez, 2012; Grupstra et al., 2021; Parker, 1984) will likely yield many undiscovered species and possibly even novel genera (Yorifuji et al., 2021). These efforts should not be limited to subtropical and tropical waters, as Symbiodiniaceae have been reported in more temperate locations (LaJeunesse et al., 2021; Lien, Fukami & Yamashita, 2012). Systematic and wide-ranging effort to better describe the genetic diversity of Symbiodiniaceae (such as the Tara Oceans expedition; Sunagawa et al., 2020) will lead to a better understanding of the drivers of taxonomic and functional diversity of Symbiodiniaceae.

### What steps can be taken to enhance our understanding of Symbiodiniaceae species?

Expanding publicly accessible Symbiodiniaceae culture collections can drive not only taxonomic but also ecological, physiological, and genomic research (LaJeunesse et al., 2018; Voolstra et al., 2021b; Xiang et al., 2013). Most of the diversity in culture constitutes just a handful of species, predominantly from the *Symbiodinium* and *Breviolum* genera. More targeted and consistent funding to support further development, maintenance, and sharing of culture collections is critical to the field. Progress toward protocols for Symbiodiniaceae cryopreservation can help conserve biodiversity through the generation of cryogenic archives (Di Genio et al., 2021) and support research in laboratories that cannot maintain continuous cultures. Depositing live specimens in national and organizational archives can alleviate the burden on individual research groups. Examples of national archives include the Provasoli-Guillard National Center for Marine Algae and Microbiota at Bigelow Laboratory in the USA, (https://ncma.bigelow.org/), the Symbiont Culture Facility at the Australian Institute of Marine Science in Australia (https://www.aims.gov.au/), the National Institute for Environmental Studies, (https://mcc.nies.go.jp/) and Biological Resource Center at National Institute of Technology and Evaluation (https://www.nite.go.jp/nbrc/catalogue/) in Japan, the Central Collection of Algal Cultures in Germany (https://www.uni-due.de/biology/ccac/), the Roscoff Culture Collection in France (https://roscoff-culture-collection.org/), and the Culture Collection of Algae and Protozoa in the United Kingdom (https://www.ccap.ac.uk/).

Live cultures established from single cells can benefit taxonomic studies by providing relatively homogeneous strains to establish baselines of diversity and morphology. Monocultures can be confirmed molecularly through fragment analysis of microsatellites. As they are haploid, Symbiodiniaceae monocultures should only show single microsatellite peaks, except in taxa with evidence for broad duplications, such as *Durusdinium trenchii* (LaJeunesse et al., 2014). Molecular data from cultured isoclonal strains are less noisy than data from host tissues, since such tissues may contain multiple Symbiodiniaceae genera, species, or strains (Fig. 1; Voolstra et al., 2021b). Cultures are also superior for holotype depositions, and they facilitate morphometric analysis, for example, on swimming behavior (motility). However, live culture is not a prerequisite for formal species description, especially because many Symbiodiniaceae are currently difficult to culture (Krueger & Gates, 2012). Furthermore, many strains cultured from host tissue do not represent the dominant Symbiodiniaceae in a host species (Santos, Taylor & Coffroth, 2001). We encourage efforts toward testing new media and bringing new species into culture (Nitschke et al., 2020), as well as documenting and sharing successful and failed attempts. “Culturability” itself may be a useful phenotype to track, as it may reflect the degree of host-specificity, and influence media or antibiotic choice (Ishikura et al., 2004; Nitschke et al., 2020; Reimer et al., 2010; Yorifuji et al., 2021). Motility, cell division rates (growth), bacterial communities (microbiomes) and viral consortia (viromes) are also informative characteristics that can vary within and among symbiont species (Grupstra et al., 2022a; Lawson et al., 2018; Levin et al., 2017; Parkinson & Baums, 2014; Yamashita & Koike, 2016). Constructing a global phenotypic database for cultures, much like the Coral Trait Database (Madin et al., 2016) is another priority for Symbiodiniaceae research, as is exploring the culturable fraction of coral-associated bacteria that may interact directly with Symbiodiniaceae and impact their performance (Frommlet et al., 2015; Lawson et al., 2018; Matthews et al., 2020; Sweet et al., 2021; Li et al., 2022).

Finally, it would be advantageous to identify and culture model Symbiodiniaceae lineages to test species boundaries. For example, measuring DNA sequence differences between sibling species separated by a geological barrier (*e.g.*, the Isthmus of Panama; LaJeunesse et al., 2018; Pochon et al., 2006) would provide molecular-divergence cutoffs that could then be applied to better resolve sympatric lineages. Additionally, cultures of closely related, putative sibling species could be used to explore cytological evidence for sexual recombination (Figueroa, Howe-Kerr & Correa, 2021), evaluate potential hybridization (Brian, Davy & Wilkinson, 2019), and characterize the role symbiotic interactions play in genome evolution (González-Pech et al., 2019).

## Guidance for population-level assessment of symbiodiniaceae

### How can we design population-level studies?

Studies evaluating the distribution of genetic variation within species, often across spatiotemporal gradients or among host taxa, seek to understand how populations are influenced by evolutionary processes such as gene flow, genetic drift, and selection (Aichelman & Barshis, 2020; Davies et al., 2020; Forsman et al., 2020; Prada et al., 2014; Reich et al., 2021; Thornhill et al., 2017; Turnham et al., 2021). Here, we define a population as a group of individuals belonging to the same species that live and interbreed with each other in a given space and time. The study of Symbiodiniaceae populations is fundamental to improving the resolution at which phenotypes of interest are differentiated. Thus, here we focus on allele-based identification and quantification of genetic variation.

Because a single host can contain a mixture of multiple species and/or genera, a first step in experimental design should include assessing sample sets for the presence of multiple distinct Symbiodiniaceae that may confound the interpretation of population-level genetic variation (see “Guidance for Community-Level Assessment of Symbiodiniaceae”). Such assessment can be done pre- and post-population-level analysis with established genetic markers (*e.g.*, ITS2, *cp23S*) and may be guided by published literature for some regions or host species. Pre-screening is especially advantageous where information on the community composition of Symbiodiniaceae is also sought and especially for hosts which tend to associate with multiple genera or species. Quantitative PCR (qPCR) is one potential technique to pre-screen Symbiodiniaceae samples for the presence of particular lineages (Correa, McDonald & Baker, 2009; Mieog et al., 2007; Saad et al., 2020). After pre-screening, population-level studies typically target genetic variation from the numerically dominant symbiont associating with a particular host or set of hosts (Baums, Devlin-Durante & LaJeunesse, 2014), while excluding any confounding genetic variation from additional species that may be present within host samples (Baums et al., 2010; Thornhill et al., 2006). Post-screening of samples is also possible using tests of assignment to genetic clusters (Davies et al., 2020) or identifying and excluding samples with outlier allelic profiles. Post-screening may be more time- and cost-effective as verification can be performed on a subset of the total sample set.

The ideal number of samples to collect and analyze will depend on the particular aim(s) of the study (*e.g.*, delineating populations *vs*. characterizing the degree of admixture among them), the scale of comparison (*e.g.*, reef, habitat, colony, intra-colony, *etc*.), and the markers being employed. However, studies leveraging more traditional markers, such as microsatellites, tend to benefit from robust sample sizes with minimum ranges of 20–30 individual hosts *per* level of interest (*e.g.*, habitat and location) (Hale, Burg & Steeves, 2012). Although this is a good target, studies limited by permit authorizations, budgets, and other constraints are still informative in some contexts.

### How can we best use microsatellite loci?

Microsatellite loci (or simple sequence repeats; SSRs) are segments of DNA where 1–6 base pairs are repeated in a tandem array; these loci are distributed abundantly across genomes of nearly all eukaryotic organisms (Tautz, 1989). Variations in the length of repeats are generated by polymerase slippage during DNA replication, resulting in homologous regions (*i.e.*, loci) of differing lengths (*i.e.*, alleles) among individuals. Microsatellites are generally thought to represent neutral loci with high mutation rates. Their single-locus, multiallelic, and codominant properties can yield valuable information regarding ploidy and reveal genetic structure among populations within and between species. Furthermore, microsatellite analyses are generally a PCR-based technique, making them cost-effective relative to other methods (Sweet et al., 2012). With the advent of high-throughput sequencing and transcriptomics, the generation of hundreds of potential microsatellite loci is now comparatively straightforward (*e.g.*, Abdelkrim et al., 2009). For species or lineages where numerous loci are available, costs and effort can remain low by multiplexing primer sets (Davies et al., 2013). Taken together, these features make microsatellites attractive for studying Symbiodiniaceae populations. These markers have been used to address questions related to overall diversity, population structure within and between reefs, gene flow, dispersal, and relatedness between symbionts (see Table 1 in Thornhill et al., 2017).

**Figure 3:**
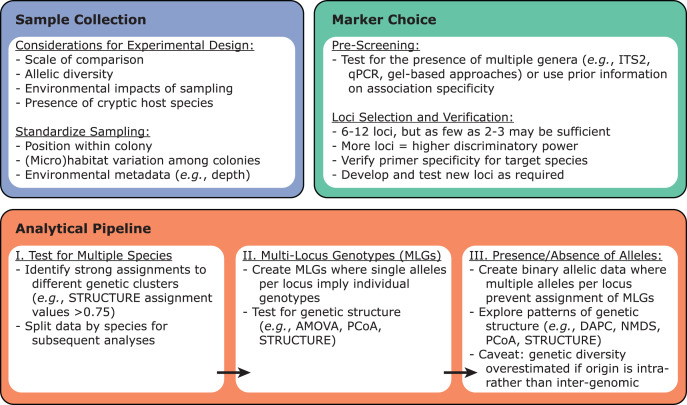
Recommendations for designing microsatellite-based Symbiodiniaceae population genetics experiments. Sample collection, marker choice, and analytical pipeline should be considered from the outset.

Once the target Symbiodiniaceae species or lineage has been identified within a dataset, these samples can be tested for variability using previously developed microsatellite loci *via* PCR amplification (Fig. 3). Primers for such loci have been developed for Symbiodiniaceae species across at least five genera: *Symbiodinium* (Pinzón et al., 2011), *Breviolum* (Andras, Kirk & Drew Harvell, 2011; Grupstra et al., 2017; Pettay & LaJeunesse, 2007; Santos, Taylor & Coffroth, 2001; Santos, Gutierrez-Rodriguez & Coffroth, 2003; Wirshing, Feldheim & Baker, 2013), *Cladocopium* (Bay, Howells & van Oppen, 2009; Davies et al., 2020; Howells, van Oppen & Willis, 2009; Magalon et al., 2006; Wham & LaJeunesse, 2016), *Durusdinium* (Pettay & LaJeunesse, 2009; Wham, Pettay & LaJeunesse, 2011), and *Philozoon* (Molecular Ecology Resources Primer Development Consortium et al., 2010). Importantly, these loci tend to have narrow phylogenetic ranges, with primers developed for a given species typically working only on other closely-related species within the same genus. Therefore, it is necessary to screen existing primers for utility with a given target species, to ensure that allelic variability among the chosen suite of microsatellite loci is sufficient, and to develop novel primer sets if existing primers fail or prove insufficiently specific. Ideally, new Symbiodiniaceae primers should be tested against monoclonal cultures of species within the same genus (positive controls) as well as against symbiont-free sperm or apo-symbiotic larvae (negative controls) to rule out off-target PCR amplification of host DNA. Although more loci will generally increase discriminatory power in population-level studies, as few as 2–3 loci have provided sufficient discriminatory power for some questions (Santos, Gutierrez-Rodriguez & Coffroth, 2003; Thornhill et al., 2009).

#### Analyses of microsatellite data

Given that Symbiodiniaceae are haploid in their vegetative life stage (Santos & Coffroth, 2003), a single allele *per* microsatellite locus is expected when a host harbors a single clonal strain of Symbiodiniaceae (represented by a single multi-locus genotype; MLG). When a single allele is recovered from nearly all loci, establishing MLGs is straightforward. However, recovery of multiple alleles at a given locus from a single sample is not uncommon (Fig. 1). Instances of multiple alleles *per* locus can be interpreted as detection of cells from multiple genetic strains (multiple MLGs) within host tissues (Andras et al., 2009; Grupstra et al., 2017; Santos, Gutierrez-Rodriguez & Coffroth, 2003; Santos & Coffroth, 2003; Thornhill et al., 2017, 2009). Examples of multiple MLGs tend to be more common within Indo-Pacific corals hosting *Cladocopium* species (Bay, Howells & van Oppen, 2009; Davies et al., 2020; Wham, Carmichael & LaJeunesse, 2014), whereas they are less common in Caribbean corals hosting *Cladocopium* and other genera (Andras et al., 2009; Grupstra et al., 2017; Pettay et al., 2015; Santos, Gutierrez-Rodriguez & Coffroth, 2003; Santos & Coffroth, 2003; Thornhill et al., 2017, 2014, 2009). Consistent patterns of multiple alleles for certain loci among a subset of monoclonal cultures has led to the proposal of whole or segmental genome duplication within certain Symbiodiniaceae. This scenario would make overestimation of symbiont genotype diversity within samples likely (Wham, Carmichael & LaJeunesse, 2014), and make the assignment of MLGs difficult, raising challenges for data analyses and interpretations.

Several approaches have been developed to accommodate instances of multiple MLGs within a sample (Fig. 3; Andras, Kirk & Drew Harvell, 2011; Davies et al., 2020; Howells et al., 2013b; Kirk et al., 2009; Magalon et al., 2006; Wham & LaJeunesse, 2016), including the exclusion of some samples and/or genotypes in certain cases. When multiple alleles for a given locus occur infrequently among samples, two data sets can be created: (1) a set where all microsatellite alleles within each sample are used and scored for presence or absence (*i.e.*, binary) within each sample, and (2) a curtailed data set omitting samples with multiple alleles at one or more loci, allowing MLGs to be assigned. Notably, studies using this approach have come to similar conclusions across the two data sets (*e.g.*, Andras, Kirk & Drew Harvell, 2011; Davies et al., 2020; Howells et al., 2013b; Kirk et al., 2009; Magalon et al., 2006; Wham & LaJeunesse, 2016). In general, given that reported scales of genetic divergence are similar across studies using binary and MLG-based approaches, and excluding many samples can lead to underestimating genetic diversity (Howells et al., 2016), we suggest that the binary approach should be used when possible (*e.g.*, a high proportion of samples exhibit multiple alleles *per* locus).

#### Caveats

While microsatellite analyses have proven informative and valuable in population genetic studies of Symbiodiniaceae, they present challenges in data acquisition and interpretation. For example, the long repetitive regions of microsatellites are often difficult to reliably amplify, making it arduous to verify repeat length *via* fragment analysis. Microsatellites can suffer from allele dropout, and low specificity of PCR primers, which can potentially lead to diversity underestimates within a sample. Microsatellites themselves are subject to more general criticisms including unclear mutation models and the potential for homoplasy (Putman & Carbone, 2014). Additionally, many analytical pipelines used to assess population genetic patterns make basic assumptions that Symbiodiniaceae do not follow (*e.g*., that organisms are diploid and exhibit predominantly sexual reproduction). In light of this, researchers should be cautious about interpreting results from pipelines developed for organisms that exhibit more traditional population biology.

### What other markers can resolve Symbiodiniaceae populations?

The ITS2 region of rDNA is repeated in tandem arrays within all known Symbiodiniaceae genomes. For population-level assessments, this universality presents an advantage over microsatellites, but the multi-copy nature of this marker poses unique challenges. As long as appropriate analytical frameworks are applied (see “Guidance for Community-Level Assessment of Symbiodiniaceae”), ITS2 data can be used to resolve strains within species. Such assessments require consideration of similarities in the assemblages of ITS2 sequences and their relative abundances within each genome. For example, genetic structure among *Cladocopium thermophilum* strains in the Persian/Arabian Gulf has been characterized (Hume et al., 2019; Smith, Ketchum & Burt, 2017) and patterns of IGV obtained from amplicon sequencing data show fine-scale spatial structure among *C. thermophilum* populations separated by tens to hundreds of kilometers (Howells et al., 2020). However, recombination (*i.e.*, whether two populations are interbreeding) is often considered sufficient for operational recognition that those entities are members of the same species (Andras et al., 2009; Grupstra et al., 2017; Santos, Gutierrez-Rodriguez & Coffroth, 2003; Santos & Coffroth, 2003; Thornhill et al., 2017, 2009). Therefore, it is difficult to determine whether ITS2-based genotypes correspond to distinct populations of the same species or different species. Other markers are also able to resolve at the population level, but their application to Symbiodiniaceae population biology is limited. Examples include the chloroplast *psbA* minicircle noncoding region (*psbA*^*ncr*^; Moore et al., 2003) and the chloroplast *23S* ribosomal region (*cp23S*; Santos, Gutierrez-Rodriguez & Coffroth, 2003).

### What are the next steps for understanding Symbiodiniaceae population biology?

Advancing our understanding of Symbiodiniaceae population biology will be greatly informed by leveraging samples that have single Symbiodiniaceae MLGs (Prada et al., 2014). For example, available monoclonal cultures of Symbiodiniaceae from several species could be used to develop and test new technologies and markers (including validation of copy number, see “Accounting for Copy Number Variation”) and these technologies could then be extended to more complex associations *in hospite* (within a host organism). To overcome the challenges of widespread gene duplication in Symbiodiniaceae genomes (González-Pech et al., 2021; Pochon et al., 2012; Prada et al., 2014), efforts should be directed toward identifying new low copy markers (or preferably single copy markers). Discovery of single copy loci may be informed by screening for universal single copy markers collated in the Benchmarking Universal Single-Copy Orthologs (BUSCO) database (Seppey, Manni & Zdobnov, 2019; Simão et al., 2015), although many BUSCOs are undetected in Symbiodiniaceae genomes (González-Pech et al., 2021). Restriction-associated DNA sequencing may serve as a low-cost method for generating single copy markers for population-level assessments in Symbiodiniaceae (Kitchen et al., 2020; Suyama & Matsuki, 2015); however, these methods require further development.

Whole-genome sequencing (WGS) is also becoming more affordable, especially at low coverage (<5X), opening the possibility of evaluating genome-wide variation in Symbiodiniaceae (González-Pech et al., 2021; Reich et al., 2021), although Symbiodiniaceae genomes are large (>Gbp) and few chromosome-scale assemblies exist (Marinov et al., 2021; Nand et al., 2021). We suggest that WGS first be applied to isoclonal cultures, where possible, to ensure reads derive from one genetic entity (Voolstra et al., 2021a; McKenna et al., 2021). Subsequently, this approach can be applied to multispecies assemblages where different Symbiodiniaceae lineages within the same genus could be mapped to these reference genomes. These types of analyses would allow for simultaneous quantification of gene flow and divergence among Symbiodiniaceae populations of co-occurring species and improve estimates of effective population sizes and clonality within and among species, hosts, and reefs. Another major advantage of genome-wide data is the potential to evaluate adaptive (non-neutral) genetic variation and signatures of selection across the genome (Ladner, Barshis & Palumbi, 2012; Liu et al., 2018; Voolstra et al., 2009). For example, identifying associations between traditional markers, genomic regions, Symbiodiniaceae functional traits, and/or environmental variables–including those that are important for the survivorship of corals under warmer, more acidic, and more eutrophic oceans–remains a research priority (see “Beyond Genotype: Phenotyping Symbiodiniaceae” and “Integrating Multiomic Technologies to Study Symbiodiniaceae”).

## Guidance for community-level assessment of symbiodiniaceae

### What is a Symbiodiniaceae community?

Generally defined, ecological communities are composed of more than one species that live together and interact. However, what is meant by terms such as “together” and “interact” can vary (Konopka, 2009), particularly when considering free-living *vs*. symbiotic Symbiodiniaceae. Typically one to two (but up to 10) Symbiodiniaceae cells reside in a coral gastrodermal cell (Davy Simon, Allemand & Weis Virginia, 2012; Muscatine et al., 1998), potentially restricting direct interactions between the endosymbiont cells within a coral host. Here, we use the term “local Symbiodiniaceae community” to refer to two or more Symbiodiniaceae species *within a single host*, whereas “macroscale Symbiodiniaceae community” (see “phenomenological community” in Konopka (2009)) describes the diversity of Symbiodiniaceae across some larger scale (*e.g.*, conspecific hosts or multiple host species). Environments that include multiple free-living Symbiodiniaceae species also constitute macroscale communities; *e.g.*, benthic sediments (Nitschke, Davy & Ward, 2016; Quigley, Bay & Willis, 2017; Sweet, 2014), the water column (Fujise et al., 2021; Porto et al., 2008), and macro-algal surfaces (Fujise et al., 2021; Porto et al., 2008).

Macroscale Symbiodiniaceae communities contain more species and encompass higher genetic diversity than local Symbiodiniaceae communities because symbiotic diversity accumulates with increased host colony and habitat sampling (Swain et al., 2020). Environmental samples include cells of symbiotic Symbiodiniaceae expelled from hosts as well as non-symbiotic, free-living species. In contrast, a given adult host typically harbors only one or two dominant Symbiodiniaceae species (Goulet, 2006), often from distinct genera, as well as other species at low relative abundances (Hume et al., 2020; Silverstein, Correa & Baker, 2012). *In hospite* Symbiodiniaceae communities can be transmitted vertically (promoting higher fidelity), reassembled horizontally (allowing for greater flexibility), or some combination of both (mixed-mode transmission) with each host generation (Quigley, Willis & Bay, 2017). The diversity of the free-living component of macroscale Symbiodiniaceae communities and the symbiotic component of local Symbiodiniaceae communities are each likely to be underestimated (*e.g.*, Baker & Romanski, 2007), but for different reasons. Free-living communities are relatively diffuse and are therefore more difficult to exhaustively sample. In contrast, local Symbiodiniaceae community assessments are prone to sampling bias (but see, *e.g*., Goulet & Coffroth, 2003). Characterizations of local communities are often based on a single sample from a well-lit, “top” surface of a colony. Sampling across a host’s surface has revealed heterogeneous distributions of dominant Symbiodiniaceae within colonies of Caribbean stony corals such as *Colpophyllia*, *Montastraea, Orbicella, Porites*, and *Siderastrea* (*e.g.*, Correa et al., 2009; Kemp, Fitt & Schmidt, 2008; Rowan et al., 1997; Ulstrup & van Oppen, 2003), as well as some Pacific stony corals (*e.g.*, Fifer et al., 2022; Innis et al., 2018; Kemp, Fitt & Schmidt, 2008; Rowan et al., 1997; Ulstrup & van Oppen, 2003) and zoantharians such as *Zoanthus* (Fujiwara et al., 2021) and *Palythoa* (Wee, Kobayashi & Reimer, 2021). Whether local Symbiodiniaceae communities exhibit structure over smaller spatial scales *in hospite* (*e.g.*, oral *vs*. aboral host surfaces) is unknown, but could be resolved with single-cell techniques (see “Integrating Multiomic Technologies to Study Symbiodiniaceae”).

### Why study Symbiodiniaceae community diversity?

Studying macroscale communities can provide insights into cnidarian-Symbiodiniaceae dynamics along environmental gradients (Cunning et al., 2015; Rossbach et al., 2021; Silverstein et al., 2011; Terraneo et al., 2019). Regional macroscale Symbiodiniaceae community structure (*i.e.*, beta diversity) may also reflect chronic disturbance from anthropogenic activity (Claar et al., 2020a) and help identify more resilient or resistant reefs (Ziegler et al., 2015). Additionally, macroscale communities in reef seawater, sediments, feces, and on macro-algal surfaces may be important sources of symbiotic Symbiodiniaceae that can be acquired horizontally by prospective hosts (Adams, Cumbo & Takabayashi, 2009; Ali et al., 2019; Castro-Sanguino & Sánchez, 2012; Coffroth et al., 2006; Cumbo, Baird & van Oppen, 2013; Fujise et al., 2021; Granados-Cifuentes et al., 2015; Grupstra et al., 2022b, 2021; Nitschke, Davy & Ward, 2016; Porto et al., 2008; Quigley et al., 2018; Quigley, Bay & Willis, 2017; Sweet, 2014; Umeki et al., 2020; Venera-Ponton et al., 2010). Symbiodiniaceae in a free-living mode may influence important processes, such as sexual reproduction, hybridization, and gene flow within Symbiodiniaceae (Figueroa, Howe-Kerr & Correa, 2021).

Positive and negative species interactions can occur within local Symbiodiniaceae communities resulting in resource and niche partitioning (Davy Simon, Allemand & Weis Virginia, 2012; Howe-Kerr et al., 2020; Matthews et al., 2020). Quantifying these interactions may help disentangle the factors and processes governing Symbiodiniaceae community assembly in early host life history stages (McIlroy et al., 2019; Quigley, Willis & Bay, 2016), as well as successional dynamics (or stability) in adult hosts. Studying local Symbiodiniaceae communities can also identify conditions that trigger symbiotic breakdown (*i.e.*, dysbiosis). Dysbiosis has frequently been documented in the bacterial communities of stressed hosts (*e.g.*, Zaneveld, McMinds & Vega Thurber, 2017; Ziegler et al., 2017; Boilard et al., 2020), and may also be evident in local Symbiodiniaceae communities. Generally speaking, dysbiosis can manifest itself in the host as: (1) an increase in symbiont richness (invasion or proliferation of low abundance symbionts), (2) a decrease in symbiont richness (loss of symbionts), or (3) more complex changes in community structure or beta diversity (Egan & Gardiner, 2016). For example, *Symbiodinium necroappetens* (LaJeunesse, Lee & Gil-Agudelo, 2015; Stat, Morris & Gates, 2008) and some symbionts in the genera *Durusdinium* (Bay et al., 2016; Manzello et al., 2018), *Breviolum* (LaJeunesse et al., 2010b), and *Cladocopium* (Wee, Kobayashi & Reimer, 2021) can opportunistically increase or decrease their abundance in bleached or stressed hosts. Stony coral juveniles in the field (Quigley, Willis & Bay, 2016) and adults in tank-based experiments (Howe-Kerr et al., 2020) have exhibited decreased survival in conjunction with more diverse local Symbiodiniaceae communities. Additional experiments to assess how frequently different types of dysbiosis occur in local Symbiodiniaceae communities are needed, including in non-scleractinian hosts, some of which can harbor up to 60 symbionts *per* host cell (Fitt, 2000). Testing the extent to which different types of dysbiosis are associated with specific cnidarian hosts, as well as specific environmental contexts, should also be prioritized.

Current challenges in understanding local Symbiodiniaceae community diversity and dynamics include: (1) determining actual and relative abundances of Symbiodiniaceae species given IGV and copy number issues (see “Accounting for Copy Number Variation”); and (2) understanding the roles (if any) that low abundance Symbiodiniaceae play in holobiont survival and fitness (see Arif et al., 2014; Bay et al., 2016; Lee et al., 2016). This knowledge is key to connecting Symbiodiniaceae genotypes to phenotypes (see “Beyond Genotype: Phenotyping Symbiodiniaceae”). Low abundance Symbiodiniaceae may serve as a reservoir of *in hospite* algal genotypes that may increase to dominance (at least ephemerally) during or following a change in environmental conditions (Bay et al., 2016; Berkelmans & Van Oppen, 2006; Boulotte et al., 2016; Buddemeier & Fautin, 1993; Claar et al., 2020a; Jones et al., 2008; Lewis, Neely & Rodriguez-Lanetty, 2019; Thornhill et al., 2006; Ziegler et al., 2018). The mechanisms controlling this turnover *in hospite* remain poorly understood, but involve host rewards and sanctions (Kiers et al., 2011, 2003) and competitive interactions among symbionts (Palmer, Stanton & Young, 2003). Competition among Symbiodiniaceae affects the initial uptake of symbionts in early coral ontogeny (McIlroy et al., 2019) and influences longer-term persistence in experimentally-generated symbioses (Gabay et al., 2019), but the relative importance of competition in shaping *in hospite* communities once they are established remains poorly understood. Beyond their potential to shift *in hospite* following bleaching events (Jones et al., 2008; Thornhill et al., 2006), low abundance Symbiodiniaceae could also contribute to emergent holobiont properties (Howe-Kerr et al., 2020; Ziegler et al., 2018). Quantification of holobiont traits with and without the addition of low abundance homologous Symbiodiniaceae (*i.e.*, lineages that typically enter into a symbiotic relationship with a given host taxon) from a range of inoculation sources constitutes a critical next step to understanding the functional role these symbionts play in the host.

### How can we optimize the study of Symbiodiniaceae community diversity?

Improving our understanding of the processes shaping Symbiodiniaceae communities is critical to predicting their distributions and potentially mitigating coral reef decline driven by global change. The methods below constitute suggested approaches for analyzing the diversity of macroscale and local Symbiodiniaceae communities. In some circumstances, identifying numerically dominant Symbiodiniaceae lineages (as opposed to the total diversity of a Symbiodiniaceae community) may be sufficient for the question at hand because hosts are generally selective in the symbionts they harbor, and some are highly specific to particular symbiont lineages (*e.g.*, Hume et al., 2020; Thornhill et al., 2014). Whether quantifying numerically dominant lineages or total Symbiodiniaceae community, the selection of molecular marker(s) and the approach(es) to data generation and analysis have implications for the interpretation of diversity. Molecular markers available for assessing Symbiodiniaceae community diversity are multicopy, and thus, present the challenge of distinguishing intragenomic from intergenomic variation. Inclusion of symbiont taxa above or below the species level in the calculation of alpha and beta community diversity is problematic as these metrics are designed for species-level input. Including anything but species-level data in the calculation of these metrics can obscure patterns and lead to under- or over-estimation of diversity.

#### Markers that behave as if single copy

The Symbiodiniaceae *SSU* (*i.e.*, Murugesan et al., 2022) and *LSU* rDNA markers as well as the *cob* mitochondrial marker are multicopy but are considered to behave like single copy loci because the vast majority of copies present are a single sequence. The few intragenomic sequence differences that do occur tend to be relatively straightforward to resolve in the context of identifying the dominant Symbiodiniaceae lineage within each genus. Many analysis algorithms produce amplicon sequence variants (ASVs), which are statistically inferred based on sequence variation within and among samples; the degree to which ASVs represent distinct genotypes may vary by marker and Symbiodiniaceae genus. For example, *LSU* consistently resolves species within *Symbiodinium* (Lee et al., 2015), but not for all of *Breviolum* (Table 1; Parkinson, Coffroth & LaJeunesse, 2015). Thus, it is important to keep in mind that when assessing total community diversity (across multiple Symbiodiniaceae genera) with *LSU*, the number of species within certain genera may be under-represented. Despite this, markers that behave as if single copy are arguably the best option currently available for assessing total community diversity in Symbiodiniaceae as they avoid the many complications associated with interpreting variation from multi-copy markers (LaJeunesse et al. 2022).

#### Multicopy markers

Among the commonly used markers, the hypervariable chloroplast *psbA* non-coding region (*psbA*^*ncr*^) can resolve below the species level in Symbiodiniaceae (LaJeunesse, Lee & Gil-Agudelo, 2015; LaJeunesse & Thornhill, 2011; Lewis, Chan & LaJeunesse, 2019; Turnham et al., 2021; Wham, Ning & LaJeunesse, 2017), while the ITS2 region can resolve at, below, or above the species level depending on the lineage. Higher resolution comes at a considerable cost in terms of complexity of analyses.

***psbA***^***ncr***^**:** The *psbA*^*ncr*^ region can assess relatedness only among closely related Symbiodiniaceae lineages within the genus (LaJeunesse & Thornhill, 2011; Thornhill et al., 2014). It is helpful to have *a priori* knowledge of the genera being amplified when using this marker (see “When should Researchers use Multiple Symbiodiniaceae Genetic Markers for Community-level Analyses?”) as available primers have known biases for specific genera. For example, the Symbiodiniaceae *psbA*^*ncr*^ primers 7.4-Forw and 7.8-Rev (Moore et al., 2003) preferentially amplify *Cladocopium* in samples of mixed communities, whereas the more recent psbAFor_1 and psbARev_1 do not (LaJeunesse & Thornhill, 2011). Although *psbA*^*ncr*^ is multi-copy and can exhibit IGV in some species, drawing inferences from these sequence datasets is still relatively straightforward because large genetic distances exist even between sequences from closely related species (LaJeunesse et al., 2021), similar to markers that do not present IGV. However, because the *psbA*^*ncr*^ region cannot be amplified across Symbiodiniaceae using a single set of primers, this marker is suboptimal for some types of community-level analyses, such as assessing total community diversity or beta diversity metrics. Nevertheless, it would be appropriate to pair *psbA*^*ncr*^ with other markers; *i.e.*, to resolve additional diversity within established ITS2 lineages (Noda et al., 2017; Reimer et al., 2017); and also to use this marker to verify ITS2 sequence variants generated *via* amplicon sequencing (Hume et al., 2019; Smith et al., 2020).

***ITS2:*** The ITS2 region of Symbiodiniaceae rDNA resolves many species and some subspecies (Hume et al., 2019). ITS2 has a broader application for defining lineages because one set of primers amplifies all known Symbiodiniaceae ITS2 sequences (note, however, that sequence variants only align well within-genus). These two favorable characteristics, in concert with its history of use within the field, make ITS2 a popular choice among researchers, even in situations when greater resolution might be achieved with alternative marker(s). Intragenomic sequence diversity is relatively high within Symbiodiniaceae ITS2 (Arif et al., 2014; Gong, Zhang & Li, 2018; LaJeunesse et al., 2022) and along with copy number, varies considerably across genera (Saad et al., 2020) and likely species (though no data are currently available at this resolution). This IGV severely restricts the inferences that can be made regarding the relative abundance of community members in cases of multiple Symbiodiniaceae lineages *per* host (see “Accounting for Copy Number Variation”). The central issue in using ITS2 to characterize symbiont diversity *in hospite* is differentiating intragenomic sequence variants (those that reflect differences within one genetic entity) from intergenomic sequence variants (those that reflect differences between two or more genetic entities). This is of particular importance because, unlike with *psbA*^*ncr*^, Symbiodiniaceae ITS2 intragenomic distances can be larger than intergenomic distances. Practically, it can be challenging to determine if sequence variation comes from one species or multiple species. Varied awareness and treatment of this issue among Symbiodiniaceae researchers has generated significant debate, which has often played out in peer review, rather than being articulated, addressed, and resolved as a research community (see “Ensuring an Inclusive Symbiodiniaceae Research Community”).

One technique to differentiate between intra- and inter-genomic sequence variants involves analyzing co-occurrence patterns. Sets of different sequences that co-occur across multiple biological replicates are more likely to be from the same genotype than to derive from multiple co-occurring lineages, with each lineage contributing a subset of the sequences. This is particularly true in cases where the relative abundances of each of the sequences of the set are similar across biological replicates. There are gel-based (Denaturing Gradient Gel Electrophoresis, DGGE; LaJeunesse, 2001) and high-throughput sequencing methods that require downstream bioinformatic analysis (*e.g.*, Frøslev et al., 2017; Green et al., 2014; Hume et al., 2019) to detect these co-occurring sequences in both dominant and low abundance taxa. Gel-based and *in silico* approaches each have their advantages and disadvantages, which have been discussed elsewhere (Saad et al., 2020). Because these techniques rely on identifying banding profiles that correspond to references (gel-based) or other biological replicates (gel- and bioinformatic-based), their power to resolve diversity generally increases with access to references or further biological replicates. For this purpose, reference sets of DGGE profiles as published in the literature (*e.g.*, LaJeunesse et al., 2010a; LaJeunesse & Thornhill, 2011; Silverstein et al., 2011), or online reference databases of *in silico* profiles (*e.g.*, at symportal.org) are available to researchers. However, strong inferences can still often be made from relatively small datasets for Symbiodiniaceae taxa that are sampled multiple times in the dataset. Both techniques rely on the same biological assumption: that coral hosts commonly associate with one numerically dominant Symbiodiniaceae taxon *per* genus. In cases where this assumption does not hold–when congeneric Symbiodiniaceae co-occur in multiple biological replicates–diversity may be underestimated with multiple taxa being considered one. Identifying intergenomic and intragenomic variation is necessary for making conclusions about diversity when using multi-copy markers like ITS2. Differentiating between this variation can be challenging, particularly when dealing with less common genotypes, smaller numbers of biological replicates, lower sequencing depths, and complex communities; in these situations, sequencing of the samples in question with an additional marker may be necessary. Critically, such an additional marker must be able to resolve between the putative taxa. For example, if attempting to ascertain whether two closely related *Cladocopium* taxa (*e.g.*, within the C3-radiation) are present in a sample, *psbA*^*ncr*^ would be more appropriate than *cp23S* as the former is highly likely to resolve between such taxa (Thornhill et al., 2014), whereas the latter may or may not (Pochon et al., 2019).

#### Assessing total Symbiodiniaceae diversity

When characterizing both dominant and low abundance Symbiodiniaceae *in hospite*, three general considerations need to be made. First, Symbiodiniaceae communities can exhibit spatial structure within an individual host (*e.g.*, Correa et al., 2009; Fifer et al., 2022; Kemp, Fitt & Schmidt, 2008; Rowan et al., 1997). Second, assessment of total Symbiodiniaceae diversity is recommended with high-throughput sequencing or qPCR (genera/species present must be known *a priori* and primers specific to these must be available or designed) as these approaches provide the resolution to detect both dominant and low abundance Symbiodiniaceae. Gel- or Sanger sequencing-based methods can provide qualitative information on diversity, but lack the resolution to detect Symbiodiniaceae present at very low abundances (*i.e*., <2–11% for restriction fragment length polymorphism-based (RFLP-based) methods, (Correa, 2009); <5–30% for denaturing gradient gel electrophoresis-based (DGGE-based) methods, (LaJeunesse, Loh & Trench, 2009; Lien et al., 2007; Loram et al., 2007)). Third, all caveats for specific markers from above still apply (*e.g.*, only diversity that can be resolved can be detected, and PCR biases may occur). Markers that behave as if single copy (*e.g*., *SSU*, *LSU, cob*) are putatively well suited to characterizing total Symbiodiniaceae diversity due to their taxonomic breadth; analyses of total diversity using these markers will often be more straightforward than with *psbA*^*ncr*^ (or ITS2). Despite this, *psbA*^*ncr*^ is also a reasonable choice when investigating total Symbiodiniaceae diversity due to its apparent low(er) copy number and intragenomic richness, as long as the community diversity in question does not exceed the taxonomic range of this marker. In these limited circumstances, *psbA*^*ncr*^ may resolve lineages well because genetic distances among taxa are relatively high with this marker.

When assessing the total diversity of macroscale Symbiodiniaceae communities, it is important to consider how molecular techniques and approaches apply to ‘free-living Symbiodiniaceae’. In the broadest sense, this term refers to all cells external to metazoan (*e.g.*, coral, mollusc) or protistan (*e.g.*, ciliate, foraminifera) hosts. These cells may be found in the water column or associated with benthic substrates. ‘Transiently free-living’ refers to Symbiodiniaceae cells that are recently released from nearby hosts but that are not adapted to proliferate outside of hosts (Yamashita & Koike, 2013). In contrast, ‘exclusively free-living’ refers to Symbiodiniaceae species with lifestyles entirely external to hosts (Jeong et al., 2014). Although exclusively free-living Symbiodiniaceae may be detected occasionally “within” host samples, such detections can be interpreted as contamination resulting from host ingestion (rather than symbiosis establishment) or adherence to mucus (Baker & Romanski, 2007; Lee et al., 2016; Silverstein, Correa & Baker, 2012). Because ‘free-living’ (*sensu lato*) Symbiodiniaceae communities on reefs are complex mixtures of these two categories, resolving this diversity presents specific challenges (Box 2).

**Box 2:** Characterizing free-living Symbiodiniaceae community diversity.

Markers that behave as if they are single copy, as well as multicopy markers, may be applied to answering questions related to free-living Symbiodiniaceae communities. Although commonly used, primer sets for ITS2 are problematic because they result in non-target amplification of other species (*e.g.*, fungi, other dinoflagellates) present in the reef environments (Sweet, 2014; Hume et al., 2018; Nitschke et al., 2020). Despite this drawback of ITS2, other markers pose greater challenges to assessing free-living Symbiodiniaceae community diversity. Specifically, the *cp23S* marker frequently amplifies non-Symbiodiniaceae plastid-containing taxa when used in free-living systems (Nitschke unpublished data). Additionally, *cp23S*’s relatively coarse taxonomic resolution in some lineages (*e.g., Breviolum*; Parkinson, Coffroth & LaJeunesse, 2015) may not be suited to some research questions. In contrast, *psbA*^*ncr*^ operates on a narrow taxonomic breadth (see “Multicopy Markers”). Thus, although there are no issues with non-target amplifications by *psbA*^*ncr*^, multiple primer pairs would be required to amplify across all species of Symbiodiniaceae likely to be of interest; some of these primer pairs have yet to be developed.
ITS2 has its own challenges for assessing free-living Symbiodiniaceae communities because the process of looking for sets of sequences that co-occur among samples as a proxy for collapsing intragenomic variants (*e.g.*, Hume et al., 2019) is not a valid approach for free-living Symbiodiniaceae. This is because in the free-living environment, multiple Symbiodiniaceae species per genus are likely to be present in a single sample. A number of strategies exist to alleviate this problem. First, free-living Symbiodiniaceae communities, while interesting for their novel diversity, are likely to be studied alongside symbiotic Symbiodiniaceae on the same reef, allowing for recognition of symbionts in the water column that are likely derived from host expulsion. For example, Fujise et al. (2021) studied coral symbionts from the C15 and C3 radiations of *Cladocopium* and generated ITS2 defining intragenomic variant (DIV) profiles, or informative assemblies of within-sample intragenomic sequences (see Hume et al., 2019 for details). These sets of ITS2 DIV sequences were then searched for in water, macroalgae, and sediment samples from the same reef. Complete sets of sequences from the C15 and C3 profiles were successfully retrieved from water and macroalgae, however in sediments only partial DIV profiles were retrieved alongside a greater representation of sequences from additional genera (*e.g., Symbiodinium*, *Freudenthalidium*, *Gerakladium*, and *Halluxium*). It is not possible to differentiate whether these partial profiles in sediments represent novel *Cladocopium* diversity not present in corals or other hosts, or if sequencing depth was exhausted due to the greater representation of diversity across the family. A second approach, analogous to the first, leverages the high culturability of Symbiodiniaceae from free-living environments (Hirose et al., 2008; Nitschke et al., 2020; Yamashita & Koike, 2013). Of 263 Symbiodiniaceae-like single cells isolated from sands of the same reef examined by Fujise et al. (2021), 114 successfully established as novel cultures belonging to the family Symbiodiniaceae (Nitschke et al., 2020). ITS2 sequences of these isoclonal cultures were later used by Fujise et al. (2021) as reference sequences and exact matches were found within the free-living communities. Again, both of these strategies rely upon building definitive sets of ITS2 sequences from Symbiodiniaceae cells of (ideally) a clonal population of a single strain within a single species, and then querying for these ITS2 sequence sets within communities of greater complexity.
Prior to the advent of high-throughput sequencing techniques, multiple markers were PCR amplified, cloned, and Sanger sequenced when examining free-living Symbiodiniaceae communities (a method which leads to issues of interpreting inter- *vs*. intra-genomic variation). For example, ITS2 and the short hypervariable region of *cp23S* (*cp23S-HVR*) have been used to study Symbiodiniaceae communities in the water column, sediments, and in stony corals in Hawaii and the Caribbean (Manning & Gates, 2008; Pochon et al., 2010). The *cp23S-HVR* primers were selected for their high specificity for Symbiodiniaceae; although the amplicons produced by these primers are of a size amenable to high-throughput sequencing workflows (~140 bp), this sequencing approach is not cost effective for these primers because the gene region appears to have less resolving power than ITS2 (Pochon et al., 2010; Santos, Gutierrez-Rodriguez & Coffroth, 2003). New, low copy number markers that resolve diversity at or below the level of ITS2 are needed to study the diversity of free-living Symbiodiniaceae communities. *psbA*^*ncr*^ has yet to be applied to free-living communities in a high-throughput approach, but this gene region is an obvious candidate.

#### Assessing beta diversity

Beta diversity can be useful for measuring changes to Symbiodiniaceae community structure over space and time (Eckert et al., 2020; Epstein, Torda & van Oppen, 2019). Although beta diversity encompasses a range of metrics including dissimilarity, turnover, nestedness, and dispersion, it is dispersion that is most commonly used to assess Symbiodiniaceae communities (Arif et al., 2014; Claar et al., 2020b; Cunning, Gates & Edmunds, 2017; Green et al., 2014; Howe-Kerr et al., 2020; Hume et al., 2019; Quigley et al., 2014). An important consideration when analyzing Symbiodiniaceae beta diversity data is establishing whether the analysis focuses on *sequence* beta diversity (*e.g.*, amplicon sequence variant data, which typically encompass copy number and intragenomic variability below the species level), or whether the analysis focuses on *ecological* beta diversity (*e.g.*, species data). Either approach may be viable, but it is important to explicitly state which is being used, and to frame interpretations based on the potential pitfalls relevant to that approach.

#### Accounting for copy number variation

**Figure 4:**
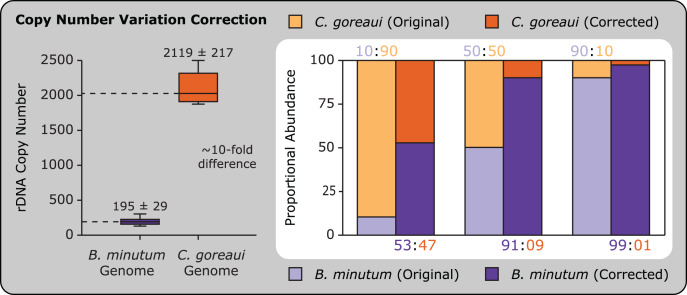
An example of ITS2 rDNA copy number variation (CNV) between the genomes of two Symbiodiniaceae species from different genera (*Breviolum minutum* and *Cladocopium goreaui*). Bar graphs demonstrate how original, uncorrected values (lighter bars) can lead to inaccurate perceptions regarding the proportional representation and numerical dominance of a species. In this case, raw *C. goreaui* ITS2 counts need to be divided by ~10 to correct for CNV (darker bars). Modified from Saad et al. (2020).

Copy number variation (CNV) is any genetic trait involving the number of copies of a gene in the genome of an individual. Efforts to quantify the absolute and relative abundances of different species in a local Symbiodiniaceae community are complicated by the presence of high CNV across taxa for key markers such as ITS2 (Correa, McDonald & Baker, 2009; Mieog et al., 2007; Saad et al., 2020; Stat, Carter & Hoegh-Guldberg, 2006; Thornhill, LaJeunesse & Santos, 2007). Thus, relative rDNA-PCR amplicon abundance does not necessarily equate to actual abundance of Symbiodiniaceae cells in a sample (especially when inter-genus comparisons are being made; Arif et al., 2014; Correa, McDonald & Baker, 2009; Quigley et al., 2014; LaJeunesse et al., 2022). For instance, some symbiont taxa that have been reported to possess a considerably higher rDNA copy number than others (*e.g., Cladocopium* spp.; Saad et al., 2020); these high copy number taxa can appear to be abundant in mixed communities even though they might represent a low fraction of cells *in hospite*, leading to inaccurate estimation of actual symbiont abundances (Fig. 4). The incorrect classification of low abundance *vs*. dominant taxa can impact interpretations related to biogeography and ecology. Such errors could be avoided if a correction factor is applied (*e.g.*, dividing the abundance value by the number of copies present in the genome of the relevant species, Fig. 4; Correa, McDonald & Baker, 2009; Mieog et al., 2007; Rubin et al., 2021; Saad et al., 2020), but such corrections rely on accurate copy number reference values, which are not currently available for the majority of Symbiodiniaceae taxa. Most studies that have quantified CNV have been limited to comparisons between genera, and there is considerable variation in the values reported across studies (*e.g.*, Gong & Marchetti, 2019; Loram et al., 2007; Mieog et al., 2007; Quigley et al., 2014; Saad et al., 2020; Thornhill, LaJeunesse & Santos, 2007). Inconsistencies in reported CNV values may be attributed to variation among strains or species (within-genus differences can be as large as between-genus differences) or to methodological differences between studies. Therefore, the extent of CNV within Symbiodiniaceae genera or across populations largely remains to be established.

Lineage-specific qPCR assays have helped to quantitatively characterize mixed communities at the genus level (Correa, McDonald & Baker, 2009; Cunning, Silverstein & Baker, 2018; Cunning & Baker, 2013) and species level (Fujiwara et al., 2021). These targeted qPCR assays are more quantitative than sequencing approaches, but still require correction for CNV. When applied to systems with known symbiont diversity, qPCR can accurately and cost-effectively quantify local symbiont community structure and dynamics; however, these primer sets must be developed on a *per*-taxon basis. Another approach is the use of flow cytometry to quantify and/or physically separate cells of interest. While the natural variability in cell characteristics (*e.g*., size, shape, fluorescence) cannot distinguish taxa (Apprill, Bidigare & Gates, 2007), the use of fluorescent probes to tag taxa of interest has successfully quantified the absolute and relative abundance of co-occurring taxa (McIlroy, Wong & Baker, 2020; McIlroy, Smith & Geller, 2014). Importantly, these methods are also conducive to subsequent genetic and physiological analyses of sorted cells. The development of further resources to account for CNV is an important priority within the field (see “Integrating Multiomic Technologies to Study Symbiodiniaceae”).

### When should researchers use multiple Symbiodiniaceae genetic markers for community-level analyses?

As each marker has its own evolutionary history and methodological bias (*e.g.*, primer bias, CNV, *etc*.), congruence among multiple independent markers should enable more robust characterization of Symbiodiniaceae community ecology (Fujiwara et al., 2021; Kavousi et al., 2020; Noda et al., 2017; Pochon et al., 2019; Reimer et al., 2017; Smith et al., 2020; Smith, Ketchum & Burt, 2017; LaJeunesse et al., 2022). Where possible, when multiple markers are used, the markers should complement each other’s taxonomic scope and power to resolve. For example, it may be productive to pair the *cp23S* (larger taxonomic scope, lower resolving power) with the *psbA*^*ncr*^ (smaller taxonomic scope, higher resolving power). Instances where multiple markers produce conflicting results may help identify a Symbiodiniaceae lineage that cannot be accurately characterized with a single broad taxonomic marker. Additionally, combining multiple markers can provide greater resolution and improved interpretability compared to single-marker approaches. For example, the use of *psbA*^*ncr*^ can help overcome issues associated with interpretation of IGV and high copy number in ITS2 and provide support for ITS2-type profiles (Smith et al., 2020) by confirming which ITS2 IGVs are most likely part of a single lineage (LaJeunesse & Thornhill, 2011; Smith, Ketchum & Burt, 2017). Budget and logistics permitting, it is recommended to use multiple markers in some situations. For some ITS2 lineages (*e.g., Breviolum* B1 or *Cladocopium* C15; Hoadley et al., 2021; Parkinson, Coffroth & LaJeunesse, 2015), available markers that behave as if single copy do not distinguish ecologically relevant variants, but universal primers have yet to be designed for gene regions that capture this variation (*e.g., psbA*^*ncr*^). Therefore, a marker that behaves as if single copy can first be applied to determine the dominant Symbiodiniaceae genera present to assist in the selection of the correct primer set for a higher resolution marker. We recognize that each additional marker can greatly increase high-throughput sequencing project costs, so such designs are only recommended when resources are available. Single marker studies can still provide great insight into symbiont community diversity as long as they are interpreted carefully.

### How can we interpret Symbiodiniaceae diversity while acknowledging the pitfalls of common markers?

Given the complexities associated with common methodological approaches and how they influence ecological interpretations of Symbiodiniaceae genetic information in community-level studies, it is critical to provide sufficient methodological details when reporting and interpreting results. We encourage the field to follow reproducible research standards, which include making analysis pipelines and raw data available after publication (see Lowndes et al., 2017 for a comprehensive guide to open science tools). At minimum, commented code (including filtering thresholds, analysis decision points, and processing steps) should be deposited in each article’s Supplemental Materials or in a publicly accessible repository (*e.g.*, GitHub) with a DOI (*e.g.*, procured through GitHub and Zenodo). Raw sequencing data must be deposited in a dedicated archive such as NCBI SRA for amplicon sequencing data, or NCBI Genbank for single sequence data. Alongside the code and sequences, additional metadata (*e.g.*, environmental and physiological parameters, as well as trackable information regarding the hosts’ ID, if applicable; Voolstra et al., 2021b), should be deposited either with the publishing journal or with a data repository (*e.g.*, Dryad, Zenodo, the National Science Foundation’s BCO-DMO). Finally, all of these deposition options can be integrated. For example, a Zenodo deposition can be linked to a GitHub repository so that new code releases are automatically updated in the Zenodo repository. By making published data widely available, we can accelerate our understanding of cnidarian-dinoflagellate symbioses and their responses to a changing environment.

There continues to be dialogue amongst members in the Symbiodiniaceae field regarding the interpretation of gene amplicon data produced by metabarcoding (*e.g.*, Illumina MiSeq). Specifically, there is an ongoing debate about if and how to incorporate Symbiodiniaceae taxa present at low abundances, and how certain parameters are built into existing analytical pipelines. For example, SymPortal will not attempt to predict profiles for a Symbiodiniaceae genus in a given sample if there are less than 200 reads for that genus/sample combination (Hume et al., 2019); this can potentially contribute to systematic underestimation of total Symbiodiniaceae community diversity. As such, we encourage authors to consider carefully what their data can and cannot discern (*e.g.*, Table 1), report assumptions associated with their data interpretation, acknowledge that other interpretations exist, and discuss whether or not these other interpretations change the biological or ecological conclusions of their study. Diversity metrics merit careful attention and gene sequence diversity should not be conflated with species diversity. Authors (and reviewers and editors) can weigh what results, tables, or figures might be included in the Supplemental Material to acknowledge and address these additional interpretations in order to facilitate the inclusion of diverse perspectives (see “Ensuring an Inclusive Symbiodiniaceae Research Community”).

## Beyond genotype: phenotyping symbiodiniaceae

### Why do we need to characterize Symbiodiniaceae phenotypic diversity?

**Figure 5:**
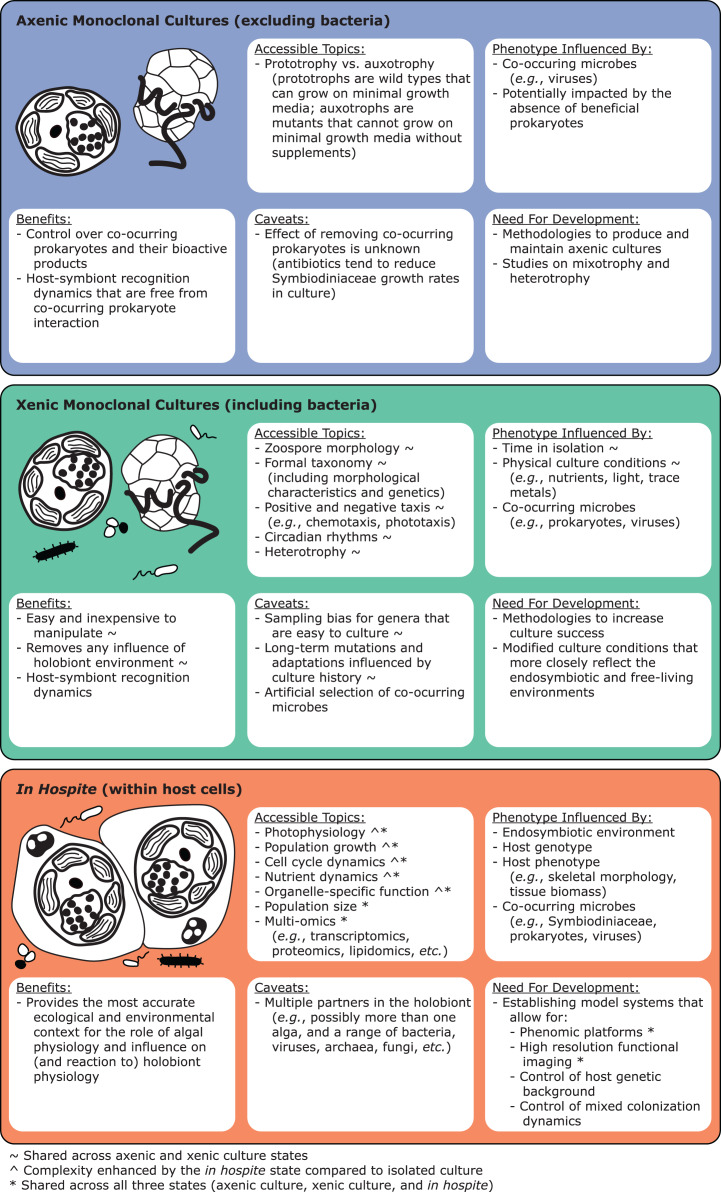
Considerations for efforts to measure Symbiodiniaceae phenotypes across three states (axenic monoclonal culture, xenic monoclonal culture, and *in hospite*).

Not all genetically distinct Symbiodiniaceae taxa exhibit physiological differences (*e.g.*, through functional convergence; Goyen et al., 2017; Suggett et al., 2015), whereas unique isolates of the same taxon may be functionally divergent (*e.g.*, Beltrán et al., 2021; Díaz-Almeyda et al., 2017; Hawkins, Hagemeyer & Warner, 2016; Howells et al., 2011; Mansour et al., 2018; Parkinson et al., 2016; Parkinson & Baums, 2014; Russnak, Rodriguez-Lanetty & Karsten, 2021). This is not surprising given the effects of strong local selection within reef habitats (Howells et al., 2011; Kriefall et al., 2022; Marhoefer et al., 2021; Suggett, Warner & Leggat, 2017; van Oppen et al., 2018) and the role of acclimatization (Torda et al., 2017). Stringent functional interrogation is therefore critical to determining how healthy cnidarian-symbiont associations will survive the climate crisis. This goal rests on advancing physiological descriptions, increasing the number of cultured isolates from diverse hosts, and extending the methodological toolbox to characterize Symbiodiniaceae differences. Thus, a more comprehensive functional characterization can accompany taxonomic assignment, helping to build greater community consensus on methods and standards for describing phenotypes of interest (Fig. 5).

### What do we need to consider when assessing Symbiodiniaceae phenotypes *in hospite*?

An overwhelming interest among Symbiodiniaceae researchers to date has been identifying thermal threshold phenotypes based on bio-optics (*e.g.*, Goyen et al., 2017; Hennige et al., 2009; Voolstra et al., 2021d), targeted biochemistry (*e.g.*, Tchernov et al., 2004) or “-omics” metrics (*e.g*., Olander et al., 2021; Roach et al., 2021). These varied foci illustrate that phenotypes are operationally defined. Consequently, the detected functional diversity (the extent and range of phenotypes resolved) may appear different depending on the metrics used. For example, descriptions of phenotype diversity for heat stress sensitivity based on photobiological properties may not align with those based on metabolic indicators (Goyen et al., 2017) or light adaptation (Suggett et al., 2015, 2022). Thus, reconciling genetic diversity with functional diversity must be carefully contextualized based on the measurement criteria and scientific questions at hand.

While it is valuable to confirm symbiont traits when in symbiosis, the presence of local Symbiodiniaceae communities and “secondary” symbionts in cnidarian holobionts complicate this effort. In local Symbiodiniaceae communities, it can be difficult to determine the relative abundance of each lineage present. Although accounting for copy number can help determine symbiont cell number and density in such cases (see “Accounting for Copy Number Variation”), other algal-centric physiological metrics, which reflect the combined average of all symbionts present within the host (Cunning, Silverstein & Baker, 2018), will be difficult to interpret. Single-cell sorting techniques may help assess unique phenotypic distinctions across different symbiont species from the same host (Snyder et al., 2020), but these techniques constitute additional effort and cost. Moreover, cnidarians host a variety of other microeukaryotic, prokaryotic, and viral symbionts (Ainsworth, Fordyce & Camp, 2017; Hernandez-Agreda, Gates & Ainsworth, 2017; Thurber et al., 2017), some of which are associated with colony health and resilience to environmental stress (Bourne, Morrow & Webster, 2016; Voolstra et al., 2021c; Ziegler et al., 2017). Some viruses even infect Symbiodiniaceae cells themselves (Grupstra et al., 2022a; Levin et al., 2017), with diverse potential impacts on Symbiodiniaceae phenotypes (Correa et al., 2021; van Oppen, Leong & Gates, 2009). The degree to which these “secondary” symbionts impact the observed phenotype of Symbiodiniaceae cells *in hospite* is an active area of research (Maire et al., 2021; Matthews et al., 2020). Finally, coral tissue thickness, pigmentation, skeletal reflectance, or other coral-associated microorganisms can affect irradiance levels reaching Symbiodiniaceae *in hospite* (Dimond et al., 2013; Dimond, Holzman & Bingham, 2012; Enríquez, Méndez & Iglesias-Prieto, 2005; Marcelino et al., 2013; Smith et al., 2013; Titlyanov et al., 2009; Wangpraseurt et al., 2014, 2012). Variation in these physiological metrics can therefore affect symbiont phenotype, and lead to variable responses to climate stress (Hoadley et al., 2019).

Traits where variability across species exceeds that within populations are ideally suited for phenotypic analysis, but are presently unknown to the field or are challenging to measure in consistent and ecologically meaningful ways. Consequently, high-throughput approaches for assessing Symbiodiniaceae phenotypes need to consider tradeoffs that are constrained by end goals. For example, recent high-throughput approaches for assessing thermal tolerance at the whole coral level—such as coral bleaching automated stress systems (CBASS; Voolstra et al., 2020)—and the single cell level (Behrendt et al., 2020) have incorporated short thermal challenges followed by stress characterization through the measurement of 1–2 physiological variables such as maximum PSII photochemical efficiency (*F*_v_/*F*_m_) and cell density. While single-phenotype assays can be informative within the context of ecosystem service values (*e.g.*, identifying thermally tolerant corals for nursery propagation; Cunning et al., 2021), identification of functionally distinct Symbiodiniaceae phenotypes will benefit from measuring a broader spectrum of physiological metrics (Hoadley et al., 2021). Phenotypic characterization using multiple photosynthetic metrics can provide some species-specific resolution (Suggett et al., 2015), and the non-invasive nature of chlorophyll *a* fluorometry lends itself to high-throughput approaches. However, poor contextualization of photosynthetic parameters with respect to cnidarian resilience currently limits the use of these techniques alone for large-scale phenomic studies, and may ultimately require integration of fitness metrics influenced by resource availability such as elemental composition *via* nutrient acquisition. While specific consensus on measurement protocols is beyond the scope of this perspective, taking a multidisciplinary approach and transparently documenting important methodological choices will help move the field forward.

### What do we need to consider when assessing Symbiodiniaceae phenotypes in culture?

Axenic or bacteria-depleted cultures are promising tools for connecting Symbiodiniaceae genotypes to phenotypes because their genetic identity is readily determined (see “Guidance for Species-Level Assessment of Symbiodiniaceae Diversity”) and morphological, physiological, and behavioral diversity are readily discernible among such algal isolates (Costa et al., 2019; Xiang, 2018; Xiang et al., 2013). In terms of photo-physiology, fluorometry has become a convenient and accessible tool to gauge “culture health” (Hennige et al., 2009; Robison & Warner, 2006; Suggett et al., 2009). Fluorometry is also used in studies examining phenotypic variation focused on photosynthetic traits and how they are affected by resource availability (light, nutrients) and temperature (Díaz-Almeyda et al., 2017; Suggett et al., 2015), and has been inferred to reflect holobiont health (Voolstra et al., 2020). While photosynthetic traits are informative of cellular functioning, they are insufficient in isolation of other measurements to explain phenotypic variation in growth (*e.g.*, Brading et al., 2011; Hennige et al., 2009; Suggett et al., 2015). Recent data point to variable photo-physiological tolerance and thermal plasticity of genetically divergent Symbiodiniaceae grown in monoculture, which has contributed to a deeper understanding of the algal symbiont response to increasing sea surface temperatures (Grégoire et al., 2017; Klueter et al., 2017; Russnak, Rodriguez-Lanetty & Karsten, 2021; Suggett et al., 2015). However, a large number of genetically distinct algal symbionts identified *in hospite* have resisted sustained growth in culture (*e.g*. Krueger & Gates, 2012; Santos, Taylor & Coffroth, 2001). Furthermore, physiological and functional ’omics data indicate that when in culture or freshly isolated, Symbiodiniaceae exhibit responses to thermal stress that differ from those of the same population *in hospite* (Bellantuono et al., 2019; Gabay et al., 2019; Goulet, Cook & Goulet, 2005). These data suggest that some physiological traits measured from culture-based studies may not be easily extrapolated to the symbiotic state. Such issues are particularly pronounced when measuring nutrient-associated phenotypes, as most culture media are nutrient-replete while Symbiodiniaceae *in hospite* appear to be nutrient-limited (Maruyama & Weis, 2021).

Emergent properties are novel characteristics that smaller units of organization gain when they become part of a larger complex system. Research focusing on core emergent properties expressed in culture, that can also be easily assessed in nature (*in hospite*), is logical given that phenotypes will consistently be the result of specific environmental conditions operating on the underlying molecular machinery. However, in decades of studies on Symbiodiniaceae cultures, the environmental conditions imposed have not consistently been reported at the time of, or prior to, sampling. Examples of such metadata include the growth phase (steady state *vs*. non-steady state; Tivey, Parkinson & Weis, 2020) or cell cycle phase (Fujise et al., 2018; Tivey, Parkinson & Weis, 2020), as well as the actual environments in the cultures (light quality/quantity, nutrients) as opposed to those measured in the incubators or assumed from the recipe of the medium used (*e.g.*, Camp et al., 2020; Reich et al., 2020), and the extent of bacterial loading. Consequently, developing guidelines for rigorous reporting of environmental (experimental) conditions when phenotypes are quantified is a key priority. Ensuring inter-comparability among studies in the future will similarly depend on operating under a more consistent set of measurement protocols for phenotypic traits.

## Integrating multiomic technologies to study symbiodiniaceae

### How can genomics and high-throughput sequencing be leveraged?

With continued cost reductions and increases in computational power and accessibility, advanced “-omic” technologies, including genomics, transcriptomics, proteomics, and metabolomics, are rapidly enhancing our ability to understand biological mechanisms (Krassowski et al., 2020). Coupled with powerful, multivariate statistical techniques and machine learning, omics technologies have greatly refined our understanding of Symbiodiniaceae biology.

Whole-genome sequencing (WGS) remains the gold standard for capturing genetic diversity (Aranda et al., 2016; González-Pech et al., 2021; Lin et al., 2015; Liu et al., 2018; Reich et al., 2021; Shoguchi et al., 2018, 2013). While individual and concatenated genes can help resolve phylogenetic relationships and define taxonomic lineages (LaJeunesse et al., 2018; Parkinson, Coffroth & LaJeunesse, 2015), WGS data provide more comprehensive phylogenomic signals, which can be used to investigate divergent selection. For example, while a Symbiodiniaceae phylogeny reconstructed using *k*-mers (short, sub-sequences of defined length *k*) derived from whole-genome sequences is largely consistent with the phylogeny reconstructed with *LSU* rDNA data (González-Pech et al., 2021), different genomic regions exhibit distinct phylogenetic signals (Lo et al., 2022). Further comparison of WGS data indicate that the similarity shared between different species within the genus *Symbiodinium* is comparable to that between different Symbiodiniaceae genera, revealing more extensive divergence than anticipated and suggesting a need for future revision (Dougan et al., 2022).

WGS efforts employ short- or long-read technologies, or a combination of both strategies. Short-read sequencing technologies (*e.g*., Illumina) have typically offered a cost-effective approach for deep sequencing with low error rates (<1%) and have provided valuable insights into Symbiodiniaceae diversity, including gene family expansions across different Symbiodiniaceae lineages (Aranda et al., 2016; Lin et al., 2015; Liu et al., 2018). Though short-read sequencing can identify lineage-specific divergence, short reads are difficult to assemble, especially with highly repetitive genomic regions. Long-read sequencing technologies such as Pacific Biosciences (PacBio) or Oxford Nanopore Technologies offer a viable alternative to improve the contiguity of highly fragmented short-read genomes. However, long-read sequencing technologies are more expensive and error-prone relative to short-read platforms, though these technologies are rapidly advancing (Karst et al., 2021). Long-read data allow us to observe chromosome structure, a greater number of genomic elements, such as DNA transposons, long terminal repeats, or chromosomal enrichment for genes with similar biological functions (González-Pech et al., 2021; Li et al., 2020; Nand et al., 2021). Chromosome-level assemblies represent a major milestone in dinoflagellate genomics as they confirm that many genes are encoded in unidirectional clusters which correspond to large topological domains (Marinov et al., 2021; Nand et al., 2021). Not only does this discovery provide insights into the structure of genes in Symbiodiniaceae, but the structure can be correlated with the encoded genes and their expression patterns to observe their interactions, elucidating novel insights into the evolution of diverse Symbiodiniaceae lineages at the chromosome level (Lin, Song & Morse, 2021).

Efforts are underway to expand the number of high-quality short- and long-read assemblies for cnidarian-associated and free-living Symbiodiniaceae and incorporate these data into taxonomic descriptions (Dougan et al., 2022; McKenna et al., 2021; Voolstra et al., 2021b). Additionally, the two read types can be coupled (*e.g.*, Illumina with PacBio HiFi) to incorporate both the contiguous sequences (>20 kb) of long reads with the low error rate of short reads, allowing robust comparisons of sequence divergence within and across genomes (Ebert et al., 2021). Once more data become available, it may be feasible to incorporate whole-genome information into future taxonomic and systematic revisions to the family (Dougan et al., 2022). However, it will be crucial to achieve consensus on how to use these data to study Symbiodiniaceae diversity and taxonomy. A lack of consistency in methodology and quality standards persists, making cross-study analyses difficult (*e.g.*, Chen et al., 2020). First steps in such standardization have been taken (Voolstra et al., 2021b; McKenna et al., 2021), but will need to be expanded as more genome data become available and the community using these data grows. Additionally, the current costs of completely sequencing the genomes of hundreds of potential Symbiodiniaceae species remains prohibitive. For the near future, feasible alternatives for WGS include using reduced representation phylogenomic approaches (such as those targeting ultraconserved elements; Cowman et al., 2020; Quattrini et al., 2020), full-length rDNA gene amplicons (Tedersoo, Tooming-Klunderud & Anslan, 2018), or entire organellar genome sequences (Liu et al., 2020). These alternatives may represent a compromise between WGS and phylogenetic marker studies by providing an intermediate amount of sequence information for taxonomic accuracy.

### How can transcriptomics, proteomics, and single-cell techniques advance our knowledge?

Researchers are keen to make functional inferences about Symbiodiniaceae, which requires focusing on coding regions. To do so requires sequencing the collection of RNAs within the cells (*i.e*., transcriptomics). Transcriptome sequencing characterizes molecular phenotypes, such as transient responses to the environment, and can reveal differential gene expression among taxa that could reflect selective pressures driving Symbiodiniaceae diversification at the functional level (Avila-Magaña et al., 2021; Bayer et al., 2012; Parkinson et al., 2016). However, the extent of gene expression changes among Symbiodiniaceae is often surprisingly subtle (*e.g.*, Barshis et al., 2014; Davies et al., 2018; Parkinson et al., 2016), although this is a matter of current debate (*e.g.*, Bellantuono et al., 2019; Voolstra et al., 2021d). Furthermore, transcription can be influenced through alternatively spliced transcripts (Lin, 2011; Méndez, Ahlenstiel & Kelleher, 2015), RNA editing (Liew et al., 2017; Mungpakdee et al., 2014; Shoguchi et al., 2020), microRNA interactions (Baumgarten et al., 2018), and methylation of mRNAs (de Mendoza et al., 2018; Lohuis & Miller, 1998; Yang, Li & Lin, 2020). These post-transcriptional modifications create variation in the transcriptome, which can complicate transcriptomic interpretation, but tracking the conservation and divergence of these variations across the Symbiodiniaceae phylogeny may elucidate novel insights into the evolution of diverse lineages.

The primary concern with bulk transcriptomic analysis is that methods often pool transcriptomes from all Symbiodiniaceae cells within a host sample, so only “average” expression profiles can be generated (Traylor-Knowles, 2021). This approach may obscure nuances in the interactions between specific symbiont and host cells, especially for less abundant symbionts. Single-cell transcriptomics (*i.e*., isolating individual cells and sequencing their transcriptomes) could solve this issue as gene expression could be explored within and among each symbiont cell *in hospite*. The generation of a cell atlas for the coral *Stylophora pistillata* has enabled the characterization of fine-scale metabolic interactions between symbionts and host gastrodermal cells (Levy et al., 2021). Single-cell sequencing can also enable high-resolution interrogations of how Symbiodiniaceae and host cells interact during symbiosis establishment, maintenance, and breakdown, particularly when Symbiodiniaceae cells can be isolated from different parts of the host coral that exhibit contrasting physiologies. By comparing expression from symbiont cells derived from different positions in the coral colony, the location and ecological role of Symbiodiniaceae can be characterized, which is a major priority for improving our understanding of symbiont communities within cnidarians.

Proteomic analyses provide an alternative mechanism to explore Symbiodiniaceae physiology and the functional impacts of different symbionts on cnidarian-algal associations, such as metabolic mismatches that occur when hosts associate with atypical (heterologous) symbionts (Sproles et al., 2019). Proteomic analyses use liquid chromatography-mass spectrometry to identify and quantify proteins, which are more directly linked to phenotype than transcript abundance (Feder & Walser, 2005). “Bottom-up” or “shotgun” approaches are commonly employed to identify and quantify as many proteins as possible in a sample in an untargeted manner, and with modern instrumentation, 3,000–4,000 proteins are commonly quantified in established model systems as well as Symbiodiniaceae (Camp et al., 2022; Richards, Merrill & Coon, 2015). Two features of proteomic analyses are particularly powerful. One is the characterization of post-translational modifications, such as protein phosphorylation, oxidation, acetylation, or ubiquitination (Witze et al., 2007). Post-translational modifications help regulate protein activity and are crucial in many biological processes, yet they cannot be detected by pre-translational analyses. The second is the ability to localize proteins to particular cellular compartments by either selectively enriching such compartments (Tortorelli et al., 2022) or fractionating the global cell content. Spatial proteomics aims to resolve the compositional architecture of cells by mapping the subcellular location of thousands of proteins simultaneously (Lundberg & Borner, 2019). Spatial resolution is achieved by separation and differential enrichment of cell content *via* density gradients or step-wise centrifugation and the subsequent quantitation of relative protein abundance across these fractions (Dunkley et al., 2004; Geladaki et al., 2019). Protein populations from differentially enriched organelles, membranes, and molecular complexes will have consistent but distinct distribution profiles and can thus be classified and mapped accordingly. Global proteome maps are powerful blueprints of the cell and can provide functional context for many uncharacterized proteins. This may be of particular use with Symbiodiniaceae, where sequence homology-based gene annotation is less effective due to their phylogenetic distance from well-studied organisms, and due to the highly derived genomes of dinoflagellates. However, these methods still require high-quality protein model search databases derived from genomic or transcriptomic sequences as they cannot identify proteins from complex samples *de novo*. Protein expression studies in combination with spatial proteomes will be powerful tools that can, for example, provide insights into the known architectural and physiological changes that accompany the symbiotic engagement of Symbiodiniaceae with their hosts. In the broader context, proteomics as a means to study the functional phenotype of Symbiodiniaceae under various conditions will likely overcome many of the current limitations of gene expression studies in dinoflagellates.

### What other omic technologies are promising?

Epigenomics, genome editing, metabolomics, and volatilomics are emerging areas within Symbiodiniaceae research. Epigenomic mechanisms, such as DNA methylation or chromatin modification, can modulate gene expression *via* gene suppression, gene enhancement, alternative mRNA splicing, or the regulation of spurious transcription without requiring any changes to genomic sequences (Bossdorf, Richards & Pigliucci, 2008; Feil & Fraga, 2012; Foret et al., 2012). DNA methylation is one epigenetic modification that occurs when methyl groups are added to DNA nucleotides, altering how transcriptional proteins bind to promoter regions thereby altering gene expression (Suzuki & Bird, 2008). Symbiodiniaceae have unusually high levels of genome methylation (Lohuis & Miller, 1998). Originally, the high level of methylation raised uncertainty about whether methylation actually played a role in gene regulation, but methylation has been linked to differential gene expression with varying irradiance (Yang, Li & Lin, 2020). Thus far, epigenomic analyses have largely been focused on the host animal, and questions are often centered around how methylation contributes to environmental tolerance (Dixon et al., 2018; Dixon, Bay & Matz, 2014; Dixon & Matz, 2021; Durante et al., 2019; Liew et al., 2018; Putnam, Davidson & Gates, 2016; Putnam & Gates, 2015; Rodriguez-Casariego et al., 2021; Rodríguez-Casariego et al., 2020; Rodriguez-Casariego et al., 2018). Therefore, determining how methylation contributes to Symbiodiniaceae functional diversity requires further exploration.

Overall, Symbiodiniaceae genomes are very difficult to annotate. At present, dinoflagellate genome and transcriptome projects rarely manage to annotate >50% of putative coding sequences *via* homology searches against genes that have been functionally characterized in other organisms (González-Pech et al., 2021; Stephens et al., 2018). In the future, genome editing could be better developed to knock out Symbiodiniaceae genes with unknown functions, making it easier to determine their biological roles. UV mutagenesis is a classic method for introducing mutations; it was used recently to create photosynthesis mutants *via* screening of colored mutants (Jinkerson et al., 2022), but its random nature is less than ideal for reverse genetics. RNA silencing is a more targeted approach that could potentially be exploited in Symbiodiniaceae studies (Zhang & Lin, 2019), but the rapidly advancing CRISPR/Cas9 technology is most desirable for its ability to knock out specific genes. Although genome editing efforts for protists have made encouraging progress (Faktorová et al., 2020), success with Symbiodiniaceae remains elusive (Chen et al., 2019; but see Gornick et al., 2022).

Biochemical analyses of Symbiodiniaceae are also in the early stages. Characterization of metabolic products (metabolomics) and volatile organic compounds (volatilomics) can provide insights into molecular cross-talk between partners. Among Symbiodiniaceae, both metabolomic and volatilomic profiles are species-specific, but they also fluctuate with environmental conditions (Klueter et al., 2015; Lawson et al., 2019; Roach et al., 2021). Distinct biochemical profiles reflect the interactions and coadaptation (or lack thereof) between the host and symbiont (Matthews et al., 2017), so biochemical assays can lead to a greater understanding of the drivers of Symbiodiniaceae evolution. As with all the omics methods mentioned so far, if metabolomics and volatilomics are to be used to understand Symbiodiniaceae divergence, more data spanning the phylogeny will be required.

### How can we integrate omic technologies?

Integrative approaches that use more than one type of technology may be required to answer intricate research questions about Symbiodiniaceae biology. For example, transcriptomics informs us of gene expression patterns, but cannot reveal protein end-products and how they are used for symbiosis, particularly because transcript abundance does not correlate well with protein levels (Cziesielski et al., 2018; Liang et al., 2021). However, when transcriptomics is integrated with proteomics or metabolomics, phenotypes can be directly observed. Then, tools that make use of multivariate statistics to combine these different types of biological data across studies, such as mixOmics (Rohart et al., 2017) or weighted gene coexpression network analysis (WGCNA; Langfelder & Horvath, 2008), can improve our ability to elucidate molecular mechanisms associated with phenotypes of interest. Thus, integration across several omic technologies (“multiomics”) holds great promise for advancing our understanding of cnidarian-algal symbiosis (*e.g.*, Camp et al., 2022), yet the financial costs associated with using multiple technologies in parallel remains a limiting factor. Additionally, the expertise of one laboratory may be restricted to one major type of analysis or instrument. Therefore, collaborations among different research groups are essential (see “Ensuring an Inclusive Symbiodiniaceae Research Community”). Logistical hurdles to collaborations include how to house and share samples and data, along with the major financial burden. Integrative approaches will remain constrained until these issues are resolved or facilitated through funding agencies.

## Ensuring an inclusive symbiodiniaceae research community

### How can we improve inclusivity in Symbiodiniaceae research?

Despite an increased recognition of the benefits of and need for more diverse representation in science, systemic biases continue to persist in science, limiting our creativity and innovation potential (Ahmadia et al., 2021). In coral reef science, where reefs are mostly found in non-industrial nations, capacity-building through “leveling the playing field” is required to facilitate a more inclusive research community and advance novel and important discoveries (O’Brien, Bart & Garcia, 2020). Marginalized groups within science have been and continue to be excluded from access to many opportunities, including funding, publishing, resources, collaborations, and networking. This exclusion is driven by limited resource availability and systemic racism, sexism, and ableism (Davies et al., 2021; Dzirasa, 2020; Ginther et al., 2011; Hoppe et al., 2019; Taffe & Gilpin, 2021; Brown & Leigh, 2018; Yerbury & Yerbury, 2021). While some progress has been made in the scientific community more broadly, there are still many deeply entrenched biases and critical gender, race, and ethnicity gaps that exist with respect to resource access; these need to be addressed by researchers, including those whose work focuses on Symbiodiniaceae, to ensure a more inclusive scientific community.

Research institutions, hiring committees, and organizers of panels, seminars, and conferences must actively work to change the demographics of scientists by increasing diversity at all career levels–from trainees to senior research scientists in positions of power. Gender, race, and ethnicity biases are rampant in the scientific-hiring process (*e.g.*, Barber et al., 2020; Bennett et al., 2020; Huang et al., 2020); for example, in the United States these biases are particularly strong against Black and Latin scholars (Eaton et al., 2020) and in New Zealand biases are stronger against people of Māori and Pasifika descent (Naepi et al., 2020). In addition to recruitment difficulties, if scholars from these backgrounds are hired, they often face continued challenges that hinder their retention. Recognizing this, people in positions of power in the Symbiodiniaceae scientific community should (1) invest in retaining a diverse workforce by promoting the academic work of minority scientists; (2) provide spaces where researchers can safely report aggressions and other challenges (Valenzuela-Toro & Viglino, 2021); and (3) create programs that provide strong multidimensional mentorship, which serve to support and retain these scholars throughout each career stage (Davies et al., 2021; Montgomery, 2017a, 2017b; Montgomery, Dodson & Johnson, 2014). Moving forward, Symbiodiniaceae researchers need to understand and implement the strategies and proposals that already exist and continue to be put forward regarding increasing recruitment and retention of historically marginalized scholars (*e.g.*, Barber et al., 2020; Chaudhary & Berhe, 2020; Greider et al., 2019). Increasing the diversity of perspectives at the decision-making table leads to more innovative discoveries (Hofstra et al., 2020; Nielsen, Bloch & Schiebinger, 2018), which are desperately needed to meet the formidable challenges of the coral reef crisis.

### How can we ensure an equitable publication process for everyone?

Equity and diversity issues exist in the scholarly publication process at multiple levels and across different areas of research. Men are first authors more often than women (Casadevall et al., 2019), notably even when both authors are identified as having contributed equally to the work (Broderick & Casadevall, 2019). Such systematic and implicit gender biases are also evident in the peer-review processes (Calaza et al., 2021). Manuscript authors, irrespective of gender, are also less likely to suggest women reviewers (Fox et al., 2017). Unprofessional reviews disproportionately impact members of underrepresented groups, who report greater self-doubt after receiving such reviews, ultimately reducing scientific productivity overall (Silbiger & Stubler, 2019). Beyond peer evaluation, more men serve in editorial roles than women (Fox et al., 2019; Grinnell et al., 2020; Hafeez et al., 2019; Palser, Lazerwitz & Fotopoulou, 2022; Pinho-Gomes et al., 2021), and editors tend to invite men more often than women to write invited reviews or perspectives. For example, the journal *Molecular Ecology*, which often publishes research from the Symbiodiniaceae community, found significant gender bias in authorship of invited ‘perspective’ articles, with women only authoring between 17.2–28.6% of these pieces (Baucom, Geraldes & Rieseberg, 2019).

Language biases are also pervasive. English is currently the default language of science, which disadvantages scientists who do not consider English as their primary language (Gordin, 2015). Non-native English speakers spend on average 97 more writing hours than native English speakers on preparation for each manuscript (Ramírez-Castañeda, 2020). In addition, ideas may be lost in translation or are often challenging to explain in a secondary language (Flowerdew, 2001). There are also costs associated with publishing in a non-native language: for example, paying for translation and editorial services. Conversely, fluent English speakers publish more research articles at higher rates than non-English speakers (Taubert et al., 2021). To address English-centric journals, regional journals publish in their native languages (Bordons, 2004), but these publications are read by a smaller readership and are cited less (Di Bitetti & Ferreras, 2017), and thereby viewed as less impactful, and are less likely to be shared widely in the Symbiodiniaceae community.

General actions that can be taken within our community to ensure a more equitable publication process include: (1) increasing diversity on editorial boards, (2) increasing the diversity of invited reviewers, as well as the authors we review for; (3) promoting cost reduction strategies (as implemented in journals like *Frontiers, PeerJ*, and *PLoS One*) whereby publication fees are prorated by country or institution type, as well as other strategies that reduce editorial costs for non-native speakers (Taubert et al., 2021); (4) promoting double-blind review processes (Budden et al., 2008); (5) intentionally citing articles led by diverse colleagues (*e.g.*, from diverse gender identities and geographical areas), thereby increasing the diversity of perspectives in the field that contribute to discussion; and (6) gathering data about where and how these inequities exist and working together as a community to take actionable steps for equity in science.

### How can we avoid parachute science?

Parachute science, sometimes referred to as “helicopter” or “colonial” science, is a common practice whereby members of the scientific community from higher-income countries fail to involve local/indigenous/native people in an equitable fashion when performing research in lower-income countries (Haelewaters, Hofmann & Romero-Olivares, 2021; Stefanoudis et al., 2021). These practices tend to be more common in ecology and conservation research (de Vos, 2020), including coral reef studies. As a community, we need to understand the largely exploitative history of our discipline and avoid perpetuating it. We should stay informed regarding the history of the lands and peoples who live in the areas in which we conduct our studies. Our specific recommendations include: (1) developing laboratory manuals that include sections outlining values and best practices (including ethics approval and necessary permits); (2) adequately training students to conduct transparent research and develop equitable relationships with members of the host region; (3) supporting the establishment of long-term collaborations and exchange programs to involve local students in research; and (4) including the development of these relationships as important components of the tenure and promotion process in departments and institutions.

Importantly, researchers from institutions in high-income regions (*e.g.*, North America, Australia, Western Europe) should: (1) be sensitive to the many challenges their colleagues in lower-income regions experience, such as a lack of funding, infrastructure, and institutional support; (2) be respectful of these collaborators by treating them as peers and not as assistants, involving them in all steps of the science, and acknowledging their intellectual contributions during discussions, and; (3) be fair to these researchers and their contributions through continued involvement in planning, manuscripts, projects, and grants generated through these collaborations. Together, these strategies can facilitate a more inclusive and collaborative Symbiodiniaceae community (Armenteras, 2021; Belhabib, 2021). Importantly, integration of members of the local community provides a long-term context to the collected scientific data, such as anecdotal observations (*e.g.*, episodes of bleaching) that may be critically informative to the research.

### How might we increase accessibility and collaboration?

There is an urgent need to increase the accessibility of Symbiodiniaceae science and foster collaboration, as innovation is necessary to address the coral reef crisis. Toward this goal, we have developed a living, global database of Symbiodiniaceae researchers and their key research expertise (http://symcollab.reefgenomics.org/; Supplemental Information). The database can be queried based on topic and methodology to aid scientists in diversifying their networks. Researchers joining the database are then also invited to participate in an open Slack channel (‘Sym Slack’, https://symslack.slack.com). These resources connect and promote diverse researchers and facilitate discussions of science from different perspectives. The COVID-19 pandemic also showcased the effectiveness of virtual conferences (case in point: this perspective is the product of a virtual workshop). Maintaining hybrid conferences with reduced costs for virtual attendance along with staggered schedules and access to presentation recordings to accommodate different time zones would ensure that the sharing of scientific information is more inclusive, alongside continued efforts to drop conference charges for lower-income countries (*e.g.*, the 15th International Coral Reef Symposium in 2022). Therefore, we encourage conference organizers to facilitate virtual attendance and funding sources for those who have difficulties traveling to foster equitable networking opportunities across people from diverse backgrounds and academic stages. Additionally, incentivizing consortia of Symbiodiniaceae researchers across diverse career stages and locations and explicitly engaging researchers from marginalized backgrounds would lead to stronger capacity building and greater transfer of knowledge. Lastly, we encourage sponsors to continue expanding their funding schemes to support international collaborations. Several examples of these efforts exist, including a new grant solicitation from the United States National Science Foundation, which calls for collaborations with Brazilian scientists through the São Paulo Research Foundation. Similar schemes (*e.g.*, Deutsche Forschungsgemeinschaft) also exist to foster collaboration between researchers in developing countries. The United States Fulbright Program and the European Union Marie Sklodowska-Curie Program are other examples that support collaborations across countries. The Japanese Society for the Promotion of Science provides funding for international exchange and research for graduate students, postdoctoral scholars, and early career scientists. These types of funding mechanisms are important because they promote wealth sharing across countries, encourage collaboration while thwarting parachute science, help with international challenges including research permitting, and ultimately lead to more open sharing of data and ideas within our Symbiodiniaceae research community and beyond.

## Conclusions

Addressing (and ultimately solving) the challenges associated with the coral reef crisis is increasingly urgent as climate change accelerates. Microalgae in the family Symbiodiniaceae play a critical role in determining coral bleaching outcomes. Advancing our knowledge of the genetic diversity of these organisms, how their diversity functionally impacts coral bleaching, and how we can apply such knowledge to mitigate climate change consequences is vital. We have identified consensus approaches for studies of Symbiodiniaceae genetic diversity at the species and population levels, while recognizing several outstanding issues regarding the characterization of community diversity. We highlight key paths forward for research including exploration of the phenotypic landscape and leveraging new technologies that are broadly applied in model systems. We also emphasize the need for increased collaboration and inclusivity among Symbiodiniaceae researchers. Overall, we acknowledge the dire need for advancing our understanding of Symbiodiniaceae ecology, physiology, and evolution, which will have the potential to expedite restoration practices and facilitate management decisions as we continue to push for political action on climate change.

## Supplemental Information

10.7717/peerj.15023/supp-1 Supplemental Information 1 Current list of formally described Symbiodiniaceae species and associated diagnostic information. Mito = Mitochondrial; Chloro = Chloroplast; R = Resolves all species within the genus; D = Diagnostic (uniquely differentiates a particular species of the genus); ND = Not diagnostic (sequence/trait identical in two or more species); M = Measured but lacking congenerics or reference material for comparison; X = Not used in species description; U = Unknown (*e.g*., sampled from a symbiotic habitat but not necessarily likely to be the numerically dominant symbiont); Y = Yes; N = No; NA = Not Applicable; ND* = Not diagnostic of species, but lack of elongated amphiesmal vesicles is diagnostic of Cladocopium; D** = Some ITS2 sequences may be diagnostic, but others in the in the same genome may not be; Y*** = Opportunistic and occurring at background levels unless host health is compromised. Compared to Table 1, this supplement also includes authentic cultured strains, synonyms, and key references for each species.

10.7717/peerj.15023/supp-2 Supplemental Information 2 Symbioidiniaceae Collaborative Community spreadsheet (as of April 18 2023).

## Additional Information and Declarations

### Data Availability

The following information was supplied regarding data availability: This is a literature review.
